# Mutations in glycyl-tRNA synthetase impair mitochondrial metabolism in neurons

**DOI:** 10.1093/hmg/ddy127

**Published:** 2018-04-10

**Authors:** Veronika Boczonadi, Kathrin Meyer, Humberto Gonczarowska-Jorge, Helen Griffin, Andreas Roos, Marina Bartsakoulia, Boglarka Bansagi, Giulia Ricci, Fanni Palinkas, René P Zahedi, Francesco Bruni, Brian Kaspar, Hanns Lochmüller, Kym M Boycott, Juliane S Müller, Rita Horvath

**Affiliations:** 1 Wellcome Centre for Mitochondrial Research, Institute of Genetic Medicine, Newcastle University, NE1 3BZ Newcastle upon Tyne, UK; 2 The Research Institute, Nationwide Children’s Hospital, Columbus, OH 43205, USA; 3 Leibniz-Institute für Analytische Wissenschaften-ISAS-e.V., Dortmund 44139, Germany; 4 CAPES Foundation, Ministry of Education of Brazil, Brazil; 5 Department of Clinical and Experimental Medicine, University of Pisa, Pisa 56126, Italy; 6 Institute of Neuroscience, Wellcome Centre for Mitochondrial Research, Newcastle University, NE2 4HH Newcastle upon Tyne, UK; 7 Department of Biosciences, Biotechnologies and Biopharmaceutics, University of Bari Aldo Moro, 70121 Bari, Italy; 8 Department of Neuroscience, Molecular, Cellular, and Developmental Biology Graduate Program and Integrated Biomedical Science Graduate Program, College of Medicine, The Ohio State University, Columbus, OH 43210, USA; 9 Department of Neuropediatrics and Muscle Disorders, Medical Center – University of Freiburg, Faculty of Medicine, Freiburg 79160, Germany; 10 Centro Nacional de Análisis Genómico (CNAG-CRG), Center for Genomic Regulation, Barcelona Institute of Science and Technology (BIST), Barcelona 08028, Spain; 11 Department of Genetics, CHEO Research Institute, University of Ottawa, K1H 8L1 Ottawa, Canada

## Abstract

The nuclear-encoded glycyl-tRNA synthetase gene (*GARS*) is essential for protein translation in both cytoplasm and mitochondria. In contrast, different genes encode the mitochondrial and cytosolic forms of most other tRNA synthetases. Dominant *GARS* mutations were described in inherited neuropathies, while recessive mutations cause severe childhood-onset disorders affecting skeletal muscle and heart. The downstream events explaining tissue-specific phenotype–genotype relations remained unclear. We investigated the mitochondrial function of *GARS* in human cell lines and in the Gars^C210R^ mouse model. Human-induced neuronal progenitor cells (iNPCs) carrying dominant and recessive *GARS* mutations showed alterations of mitochondrial proteins, which were more prominent in iNPCs with dominant, neuropathy-causing mutations. Although comparative proteomic analysis of iNPCs showed significant changes in mitochondrial respiratory chain complex subunits, assembly genes, Krebs cycle enzymes and transport proteins in both recessive and dominant mutations, proteins involved in fatty acid oxidation were only altered by recessive mutations causing mitochondrial cardiomyopathy. In contrast, significant alterations of the vesicle-associated membrane protein-associated protein B (VAPB) and its downstream pathways such as mitochondrial calcium uptake and autophagy were detected in dominant *GARS* mutations. The role of VAPB has been supported by similar results in the Gars^C210R^ mice. Our data suggest that altered mitochondria-associated endoplasmic reticulum (ER) membranes (MAM) may be important disease mechanisms leading to neuropathy in this condition.

## Introduction

All genes are copied into short-lived RNA molecules, which are then translated to proteins, forming the building box of the cells in the body. Although the majority of protein synthesis happens in the cytosol, an additional translation apparatus is required to translate 13 proteins important in mitochondrial energy production, which are encoded by the mitochondrial genome (1). The majority of genes which regulate protein translation in these cellular compartments are distinct, but two genes encoding aminoacyl-tRNA synthetases of glycine (*GARS*) and lysine (*KARS*) are common, in both mitochondria and the cytosol (2). Investigating the function of these bi-functional enzymes provides a unique opportunity to explore interactions between cytosolic and mitochondrial translation and facilitates the dissection of tissue-specific manifestations of mitochondrial or cytosolic defects of translation.

**Figure 1.**
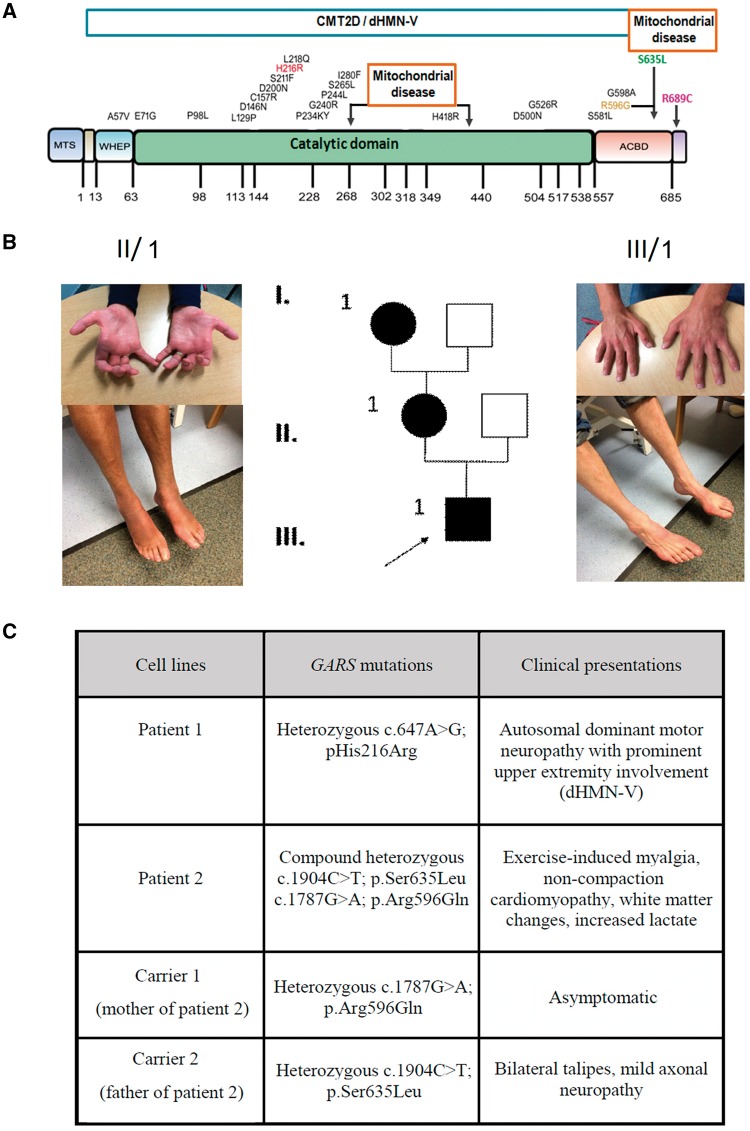
Schematic representation of the GARS protein and distribution of dHMN-V and mitochondrial disease-associated dominant and recessive mutations. (**A**) Dominant mutations causing CMT2D/dHMN-V are mostly located in the catalytic domain marked with black. Recessive mutations leading to mitochondrial disease localized in the catalytic domain and at the anticodon binding domain (ACBD) shown by the black arrows. Mutations modelled in this study are highlighted with red, orange, green and purple. (**B**) Pedigree of patient 1 with a novel heterozygous c.647A>G, p.(His216Arg) mutation, both patient 1 and his affected mother show prominent atrophy of small hand muscles and moderate atrophy and weakness in the feet. (**C**) Summary of the clinical presentations of the patients (patient 1 and 2) and heterozygous parents of patient 2 (carrier 1 and 2) whose fibroblasts were used in this study.

*GARS* encodes the non-redundant homodimeric glycyl-tRNA synthetase, covalently attaching glycine to its cognate tRNA, which is essential for the fidelity of protein translation (2). There are two translation initiation sites resulting in the production of mitochondrial and cytoplasmic isoforms of *GARS*. Three functional domains are common in both isoforms: a highly conserved N-terminal WHEP-TRS domain, a catalytic core and a C-terminal anticodon-binding domain, however a mitochondrial targeting signal (MTS) is only present in mitochondrial *GARS* (Fig. 1A). Autosomal-dominant *GARS* mutations cause axonal CMT (CMT2D) or distal hereditary motor neuropathy with upper limb predominance (dHMN-V) (2). While the majority of CMT2D mutations were localized to the catalytic domain, autosomal recessive mutations were reported in three independent patients with a mitochondrial phenotype (Fig. 1A). One baby boy, homozygous for c.2065C>T, p.(Arg689Trp), had severe neonatal cardiomyopathy and cytochrome *c* oxidase deficiency and died at 10 days of age (3). Another child with compound heterozygous c.1904C>T, p.(Ser635Leu) and c.1787G>A, p.(Arg596Gln) presented with exercise-induced myalgia, non-compaction cardiomyopathy, periventricular lesions and increased lactate (4). Recently, recessive mutations within the catalytic domain were also reported causing multisystem disease with growth retardation, delayed motor milestones, dysmorphic signs and complex neurological presentation of microcephaly, thinning of the corpus callosum, white matter lesions, cerebellar vermis and brainstem atrophy, but without peripheral neuropathy (5). To date no neuropathy was observed in children with recessive mutations however it may develop later in life. A mild neuropathy was observed on electrophysiological testing in the heterozygous father of the second child (4).

Reduced aminoacylation activity, altered axonal localisation (6,7), impaired catalytic function (8) and more recently the altered neuropilin 1 pathway (9) were described to contribute to the disease in *GARS* mutations. However, to date, little is known about the mitochondrial role of *GARS* and how it affects the disease phenotype. Thus, in this study, we explored the mitochondrial function of the bi-functional enzyme and show that mutations in *GARS* lead to tissue-specific mitochondrial defect in neurons with different pathomechanism in autosomal dominant and recessive mutations.

## Results

### Patients

We studied cell lines of patients carrying pathogenic *GARS* mutations (Fig. 1B and C).

Patient 1 and his mother (II/1) manifested with typical clinical and electrophysiological signs of dHMN-V, both developed upper limb predominant distal neuropathy since early twenties (Fig. 1B). They both carry the c.647A>G, p.(His216Arg) mutation in *GARS*, detected on a diagnostic multigene next generation panel. This variant is predicted to be pathogenic based on Association for Clinical Genetic Science (ACGS) Practice Guidelines (2013) and segregated with the phenotype in the family The clinical presentation and family history of patient 2 have been reported previously (4). The 12-year-old girl developed cardiomyopathy and exercise intolerance at age 6 and carries the compound heterozygous c.1904C>T, p.(Ser635Leu) and c.178G>A, p.(Arg596Gln) *GARS* mutations. The mother is heterozygous for the c.1787G>A, p.(Arg596Gln) mutation and shows no signs of a neuropathy, the father has a mild neuropathy and carries c.1904C>T, p.(Ser635Leu).

### GARS is localized to mitochondrial RNA granules

**Figure 2.**
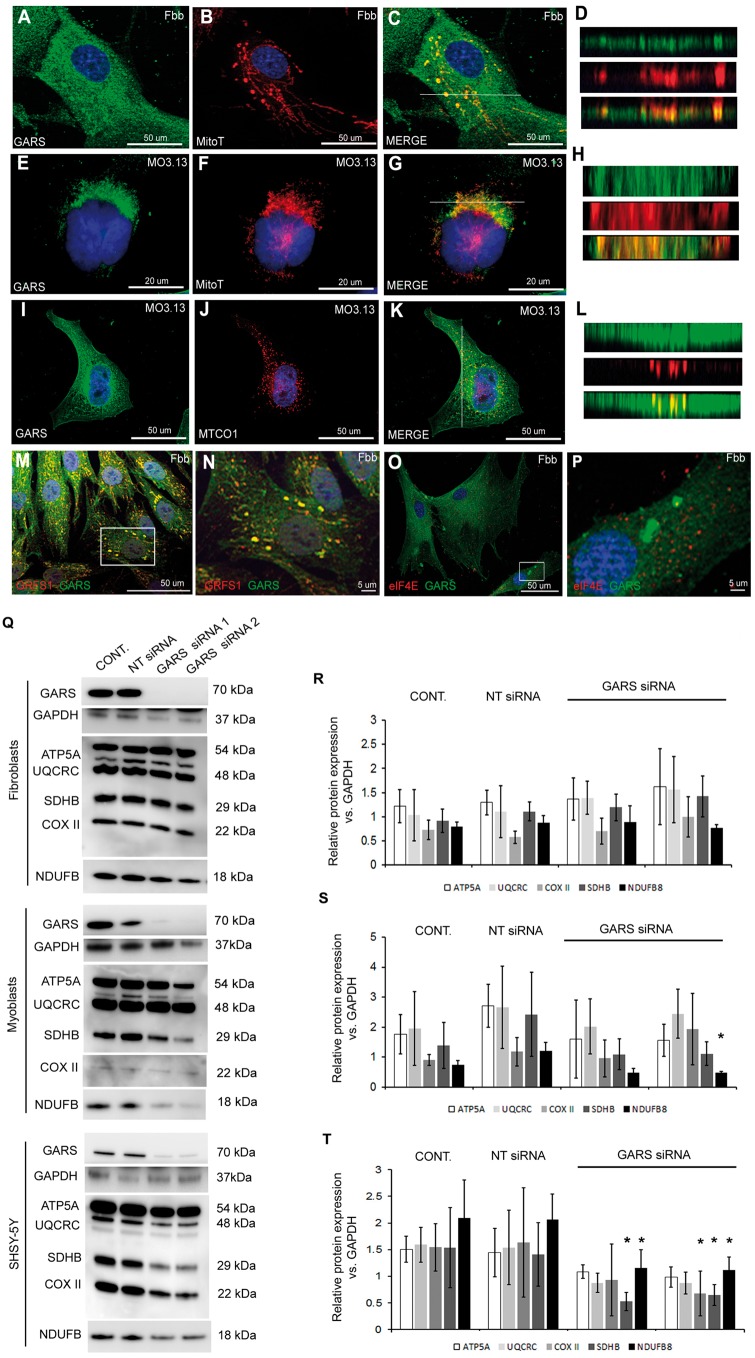
Endogenous GARS downregulation in human cells. (**A**) Human control fibroblasts and (**E**, **I**) MO3.13 cells were immunostained for GARS (green) and mitochondria (Mitotracker, red) (**B**, **F**, **J**). Merged images are shown in (**C**, **G**, **K**). White lines indicates the location of the XY slice. Representative XY sections show co-localization of GARS with mitochondria (Mitotracker) in fibroblasts (**D**) MO3.13 cells (**H**) and GARS-MTCO1 (red) co-localization in MO3.13 cells (**L**). Staining for the mitochondrial RNA granule marker GRFS1 (red) and GARS (green) in fibroblasts (**M**). Boxed area is shown as zoomed image (**N**). Staining for cytosolic RNA granule marker eIF4E (red) and GARS (green) in fibroblasts (**O**). Boxed area is shown as zoomed image (**Q**). Human fibroblasts, myoblasts and SHSY-5Y cells were subjected to siRNA mediated *GARS* downregulation. Cells were transfected with non-targeting (NT) siRNA, and two different *GARS* siRNAs (*GARS* siRNA 1 and 2) for 9 days. After 9 days of treatment total cell lysates were collected from the cells. The silencing of GARS protein and the steady-state level of mitochondrial proteins were determined by western blots. GAPDH was used as loading control (**R**, **S**, **T**). Bar graph indicates the level of mitochondrial proteins in un-transfected (control), NT siRNA transfected and *GARS* siRNA treated fibroblasts (R), myoblast (S) and SHSY-5Y cells (T). Error bars show standard deviation. Three independent experiments were performed. One-way ANOVA followed by Bonferroni *post-hoc* test (with 97% confidence intervals) were used in comparing the level of protein expression (*P < 0.5 versus cont. and non-targeted samples).

To prove that GARS is present in the mitochondria and is involved in mitochondrial translation, we performed double immunofluorescence staining (MitoTracker Red and GARS) in human fibroblasts (Fig. 2A–C) and oligodendroglia cells (Fig. 2E–G). GARS was detectable both in the cytoplasm and mitochondria (Fig. 2A–L) and was present in mitochondrial RNA granules (Fig. 2M and N), which was confirmed using multiple markers including Mitotracker, MTCO1 and GRSF1, respectively. GARS-associated aggregates in fibroblasts were previously reported (10). These cytoplasmic granules labelled with eIF4E representing processing bodies (PB) or stress granules (SG) did not confirm co-localization (Fig. 2O and P). These results show that GARS is present in RNA granules in mitochondria, thus may affect mitochondrial translation.

### siRNA down-regulation of *GARS* results in abnormal mitochondrial translation in myoblasts and oligodendroglia

We first tested *in vitro* whether mitochondrial protein synthesis in fibroblasts is altered by any of these variants. None of the fibroblasts from these patients showed a mitochondrial translation defect, which is not uncommon in mitochondrial diseases with tissue-specific clinical presentations. Next we performed siRNA-mediated downregulation of *GARS* with two different siRNAs in normal human fibroblasts, myoblasts and also in SHSY-5Y cells, providing the most relevant cellular model of GARS-related disease. We detected significantly decreased protein levels of respiratory chain subunits in myoblasts (Fig. 2Q and S) and SHSY-5Y cells (Fig. 2Q and T), but not in fibroblasts (Fig. 2Q and R), which is illustrating that loss of function of *GARS* result in a tissue-specific mitochondrial translation defect. Both siRNAs successfully down-regulated GARS in all cell types both in the cytosol and mitochondria (data not shown).

### Reduced mitochondrial respiratory chain enzymes in induced neuronal progenitor cells (iNPCs) of patients carrying dominant and recessive *GARS* mutations

**Figure 3.**
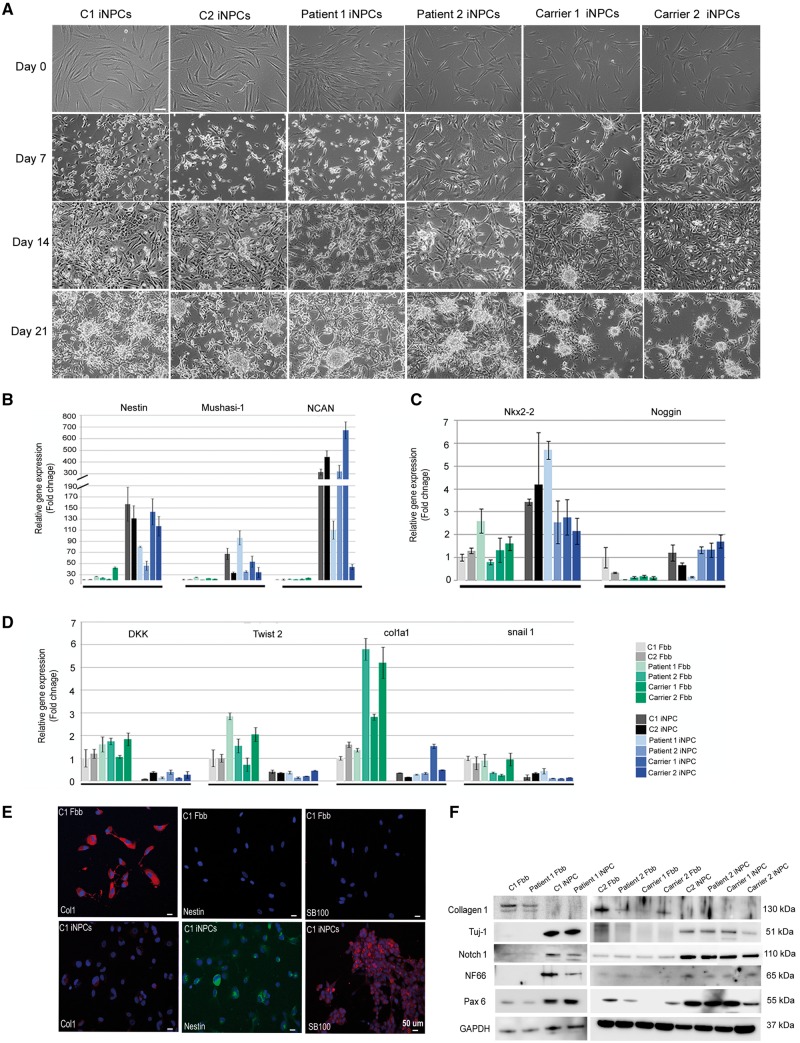
Characterization of iNPCs. (**A**) Phase-contrast images show the conversion process from the initial fibroblasts to iNPC cultures. Morphological changes were apparent after 7 days of transfection. Cells began to form sphere-like structures at 21 days. Scale bars, 50 µm. iNPCs and primary fibroblasts were collected and subjected to total RNA extraction and real-time PCR analysis. (**B**, **C**) iNPC markers (Nestin, Mushashi-1, Noggin, Ncan and Nkx2-2) demonstrated significantly higher expression levels, suggesting neuronal fate, and (**D**) reduced expression of fibroblast markers (Dkk1, Twist2, Col1a1 and Snail1) indicated loss of fibroblast characteristics (*n* = 3 independent experiment). Error bars represent standard deviation. (**E**) Immunofluorescence staining of fibroblasts and iNPC cultures at day 20 reveals loss of Collagen I (fibroblast marker) and increased expression of the iNPC markers Nestin and SB100 in the iNPCs (bottom row). DAPI was used to visualise nuclei. Scale bars, 20 µm. (**F**) Protein levels of iNPC marker proteins (Tuj-1, Notch1, NF66, PAX6) demonstrated higher levels in the iNPCs confirming the neuronal fate. GAPDH was used as loading control.

Based on the neuron-specific manifestations in patients, we studied neuronal cell lines. We took advantage of a recently developed method converting human fibroblasts directly into iNPCs to model neurodegenerative disorders (11). Within one week of conversion, the expected change in morphology was observed (Fig. 3A) and efficient viral transduction was confirmed by quantitative RT-PCR (Supplementary Material, Fig. S1A–D). Primary fibroblasts from two patients, two *GARS* mutation carriers and two healthy controls were converted (Fig. 3B) and RT-PCR (Fig. 3B–D), immunofluorescence staining **(**Fig. 3E**)** and western blot (Fig. 3F) for fibroblast and iNPC markers revealed successful down-regulation of fibroblast markers and strong upregulation of iNPC markers in all cell lines (Supplementary Material, Fig. S1E).

### RNA sequencing (RNAseq) in induced neuronal progenitor cells (iNPCs) of patients 1 and 2

**Figure 4.**
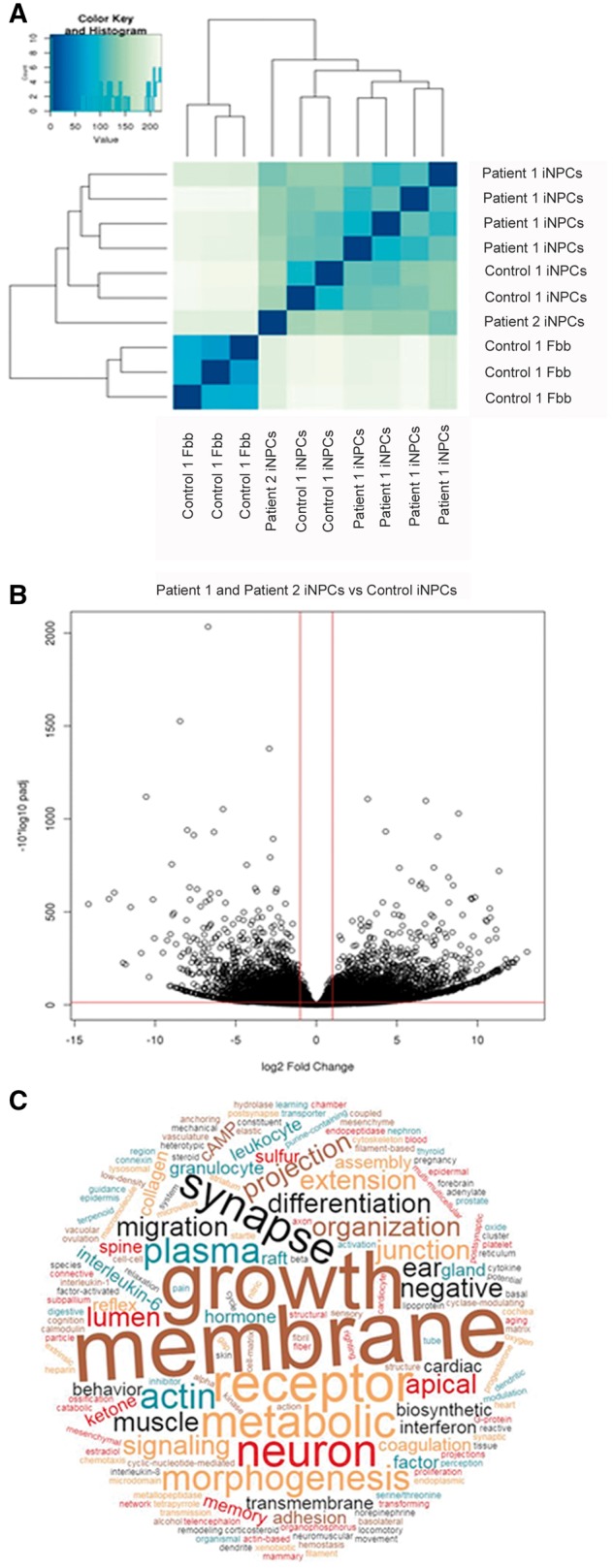
RNAseq analysis of patient and control iNPS, and control fibroblasts. (**A**) Heat map showing hierarchical clustering of the RNAseq read count sample distance matrix demonstrating the dissimilarity between the RNA sequencing expression profiles of the control fibroblasts versus the iNPCs (control and patient) (**B**) Volcano plot of −log10 adjusted *P*-value versus the log 2 fold change in RNA expression of iNPCs cells compared to fibroblasts, again demonstrating the large number of RNAs showing differential expression between the iNPCs and fibroblasts. (**C**) Word Cloud of GO terms that were significantly enriched for genes showing significant up-/down-regulation of RNAs in both patients’ iNPC cells versus control iNPC cells.

To further address the conversion toward neuronal cell linage, RNA isolated from control fibroblasts and control and patient iNPC cells, was subjected to RNAseq analysis. The iNPC cell lines of patient 1, patient 2 as well as a control iNPC line were clearly grouped together in a hierarchical clustering of the RNA read count sample distance matrix (Fig. 4A). This indicates that the iNPCs are not related to fibroblasts and they are likely to exhibit a functionally distinct and unique transcriptome. Examination of the log 2 fold change in control iNPC versus control fibroblast RNAseq read counts revealed that thousands of genes exhibited a signiﬁcant difference in expression between the fibroblast and iNPC lines (Fig. 4B). This included differential expression of genes involved in neuronal function. GO terms that were significantly enriched for genes showing significant up-/down-regulation of RNA expression in both patient iNPCs lines versus control iNPCs are represented in Figure 4C, compared with the distinct lists of the most significantly enriched GO terms, specific to each patient shown in Supplementary Material, Figure S2A and B. In further support of the neuronal fate in our iNPCs neuronal markers *NES, CD24, ROR2, FZD3, USP44* are significantly overexpressed in iNPCs compared with fibroblasts and we detected *SOX2* expression, which is not present at all in fibroblasts. These neuronal markers have been previously reported as specific for neuronal stem cells and provide evidence of a neuronal fate in our iNPCs (12). We could not detect significant changes in mRNA expression of genes of mitochondrial proteins which showed significant changes in the proteomic data, therefore we suggest that the changes occur on the protein level, which is in line with the defect in protein translation and altering protein–protein interactions.

**Figure 5.**
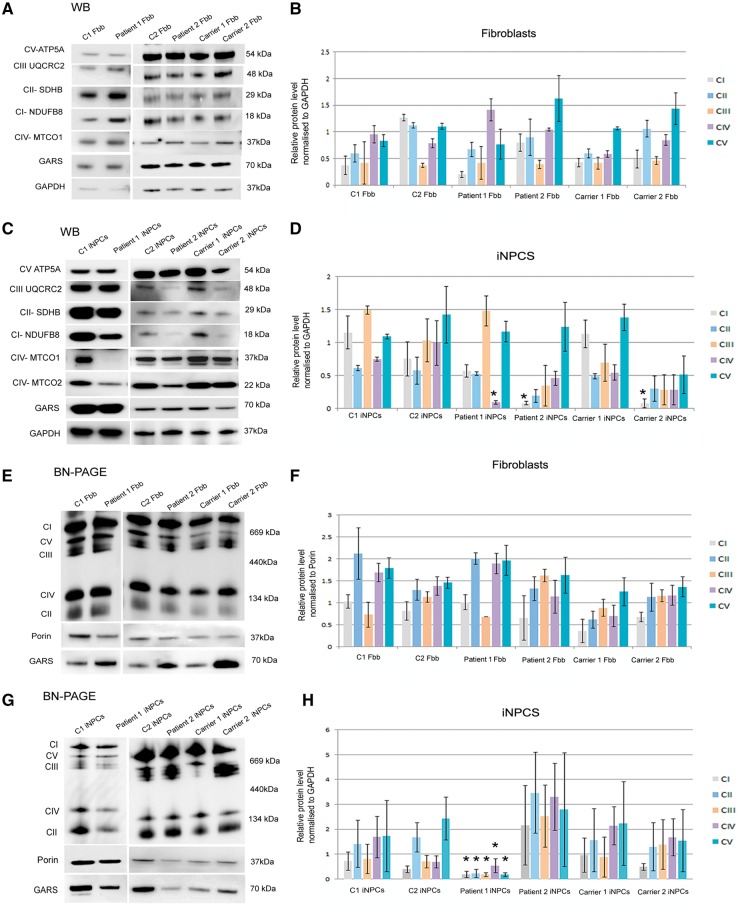
Mitochondrial protein expression and level of RC enzymes in fibroblasts and iNPCs. (**A**, **C**) Western blot analysis of fibroblast and iNPCs cell lines: two controls (C1, C2), patient 1, patient 2, carrier 1 (asymptomatic mother) and carrier 2 (father with mild neuropathy). Immunoblotting detected the expression of mitochondrial proteins. GAPDH was used as loading control. (**E–G**) BN-PAGE analysis studied the assembly and level of RC enzymes in fibroblasts (E) and iNPC cells (G). Porin was used as loading control. (**B**, **D**, **F**, **H**) Quantification of the levels of mitochondrial proteins and individual OXPHOS complexes were normalized to GAPDH and porin levels respectively. Bar graphs representing the relative mitochondrial protein expression were analysed with unpaired *t*-test on Sigma plot (version 11.0) where samples represent *n* = 3 (experimental replicates), *P*-values are from paired *t*-test: *P < 0.05. Bar graphs represents the mean ± standard deviation *P < 0.05.

After confirming the successful conversion of the cell lines, we measured mitochondrial function in iNPCs. The number of mitochondria and the copy number of mtDNA gradually increased in iNPCs and the defect became more pronounced after eight passages, confirming the higher energy demand in neuronal cells (Supplementary Material, Fig. S3A). While no defect was observed in fibroblasts (Fig. 5A and B, E and F), we detected significantly decreased level of the complex I subunit NDUFB8 in patient 2 and the mildly affected father (carrier 2), while a mitochondrial encoded complex IV subunit (MTCO1) was significantly reduced in iNPCs of patient 1 (Fig. 5C and D). Mitochondrial proteins remained normal in control iNPCs and in the healthy mother (carrier 1) carrying the heterozygous p.(Arg596Gln).

**Figure 6.**
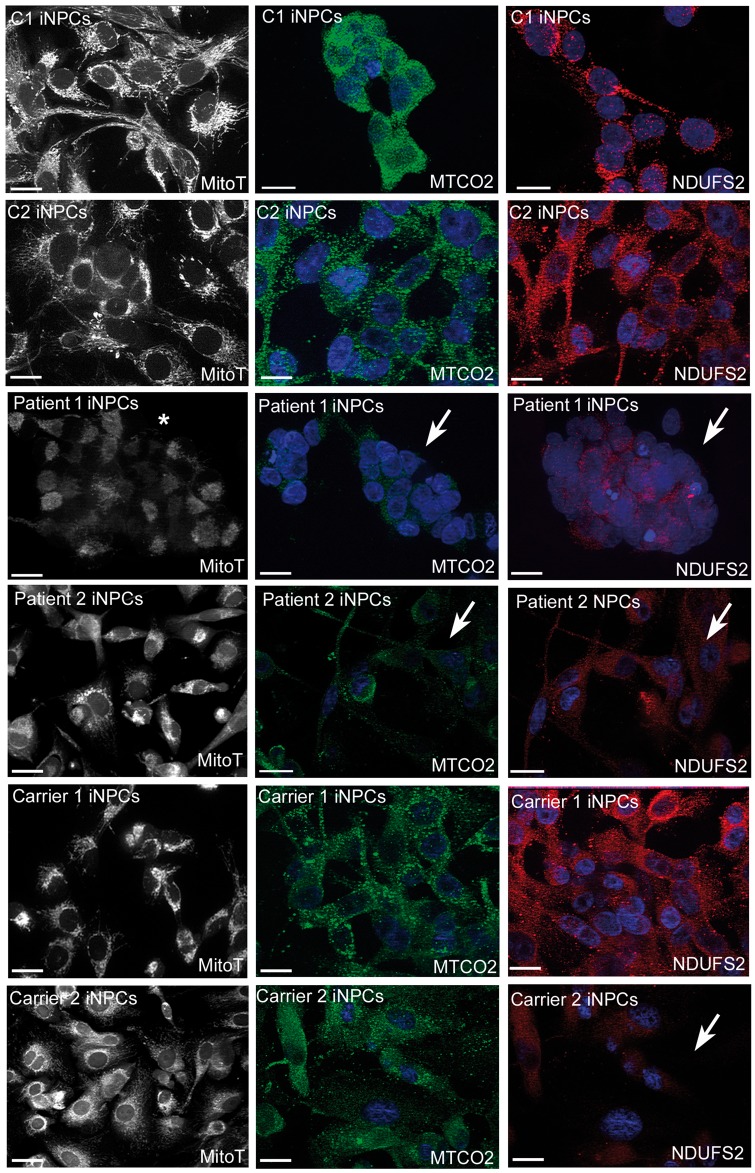
Immunostaining of mitochondrial proteins in human fibroblasts and iNPCs. Representative fluorescent images of mitotracker (black and white), MTCO2 (green channel) and NDUFS2 (red channel). Abnormal MitoTracker staining indicates abnormal mitochondrial membrane potential in patient derived iNPCs (marked with white star). iNPCs with GARS mutations in patient 1, patient 2 and carrier 2 show weak signals of mitochondrial proteins (indicated with white arrows) confirming mitochondrial translation defect (scale bar represents 20 um).

BN-PAGE showed significantly lower levels of all five mitochondrial complexes in iNPCs of patient 1, but no defect was observed in any of the other iNPC lines (Fig. 5G and H) or in fibroblasts (Fig. 5E and F). Interestingly, mitochondrial GARS was increased in symptomatic patients’ fibroblasts, suggesting compensation, possible through redistribution (as seen in mice), while in iNPCs from all patients and carriers, the amount of GARS was consistently lower in both mitochondria and cytosol (Supplementary Material, Fig. S3B–E). Immunofluorescent staining confirmed lower expression of respiratory chain subunits of complexes I and IV (NDUFS2, MTCO2) in iNPCs of all symptomatic individuals (Fig. 6) raising the possibility that the mitochondrial translation defect may be caused by the loss of function of GARS in neurons.

MitoTracker Red CMXRos accumulates only in actively respiring mitochondria that have an intact membrane potential (ΔΨm) (13). A deficient MitoTracker Red staining and the less elongated appearance of mitochondrial networks in iNPCs derived from patient 1 suggest that ΔΨm is altered in these cell lines (Fig. 6).

**Figure 7.**
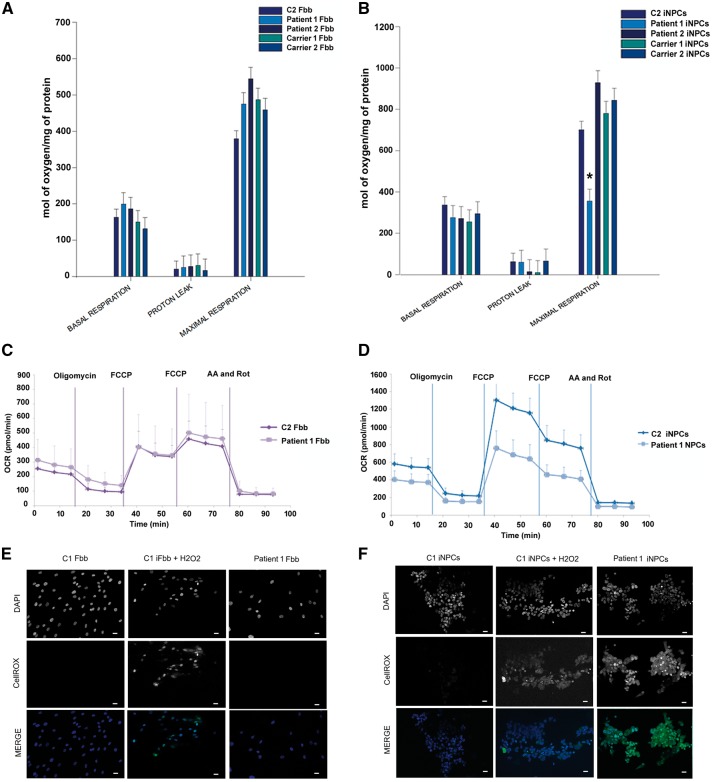
Mitochondrial functional studies. Oxygen consumption levels were monitored using the Seahorse Bioscience ExtraCellular Flux Analyser in real time. OCR is indicative of OXPHOS. Basal respiration, proton leak and maximal respiration of fibroblasts (**A**) and iNPCs are shown (**B**). Values were calculated from the mean values of each real-time run normalized to the protein concentration (*n* = 3). Oxygen consumption data are presented as ± standard deviation using two-way ANOVA test on Sigma plot (version 11.0) and paired *t*-test. A *P*-value of ≤0.05 was considered significant. The *P*-values are from an unpaired *t*-test. Where indicated *P < 0.05. (**C**, **D**) Real-time effect of control and patient 1 cells (GARS^His216Arg^) is demonstrated on OCR in fibroblasts and in iNPCs. (**E**, **F**) Fluorescent micrographs of control and fibroblasts and iNPCs of patient 1 show intracellular ROS formation as stained by the CellRox Green Reagent. Light green fluorescence indicates increased levels of oxidative stress in H_2_O_2-_treated cells as positive controls and in iNPCs of patient 1. DAPI shows nuclei. Scale bar = 20 μm.

Mitochondrial respiration measured on Seahorse XF96 showed that all fibroblasts from affected individuals with different *GARS* mutations had slightly higher maximal respiration than controls, suggesting compensation, while there was no difference in basal respiration and proton leak (Fig. 7A–D). However, we detected significantly reduced respiration in iNPCs of patient 1 (Fig. 7A–D). Additional analysis of iNPCs of patient 1 revealed increased oxidative stress (CellROX^®^ Green staining) (Fig. 6E and F).

In summary, we detected a neuron-specific defect of mitochondrial proteins in iNPCs of patient 1, which correlated with the neuropathy.

### Comparative mitochondrial proteomics identified different alterations in iNPCs of patients with dominant and recessive *GARS* mutations

**Figure 8.**
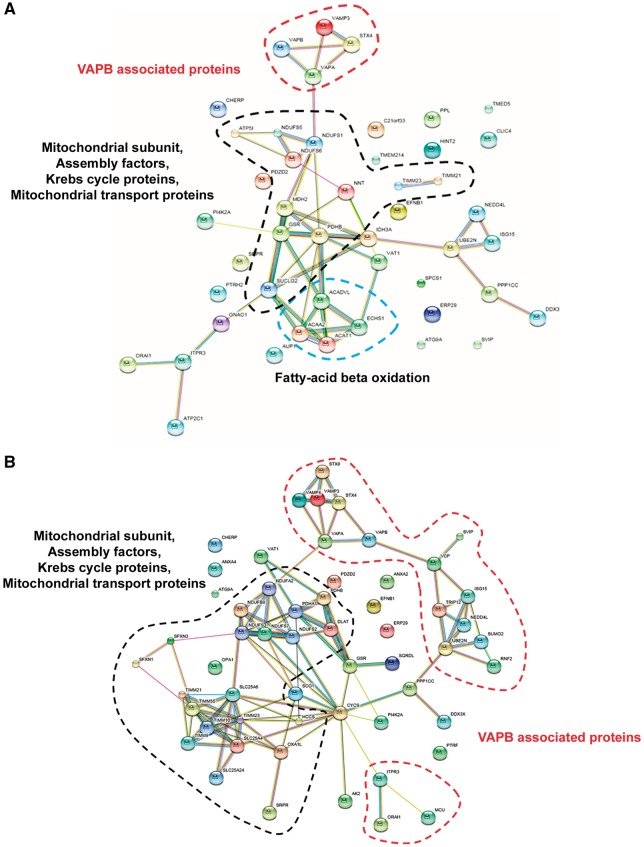
Differentially regulated mitochondrial proteins identified in patient iNPCs. Comparative proteomic analysis of mitochondrial proteins was performed on three iNPC cells lines (patient 1, patient 2 and control). A total of 85 differentially expressed mitochondrial proteins were identified in comparison between control and patient groups. Screen shot of the STRING (Search Tool for the Retrieval of Interacting Proteins) showed a network of differentially expressed proteins in cells of patient 2 (**A**) and patient 1. (**B**) Black circles indicated protein clusters directly affecting mitochondrial function. Red circles represent protein clusters related to vesicle-associated membrane proteins and its downstream pathways. Blue circle represents fatty acid oxidation pathway. The interactions are shown in confidence view. Thicker lines represent a stronger association. The proteins are identified by their gene names located near each sphere.

Comparative proteome profiling in iNPCs of patient 1 (dominant) and patient 2 (recessive) identified significant changes in several mitochondrial proteins when compared with control iNPCs. A detailed analysis of cellular processes influenced by these proteins was performed using STRING (Search Tool for the Retrieval of Interacting Proteins) software (version 10.0). The protein–protein interactions between the altered proteins analysed by STRING is shown in Figure 8A and B. We observed >2 times different abundance in both patients’ iNPCs of subunits and assembly factors of respiratory chain enzyme complexes, Krebs cycle enzymes and proteins involved in mitochondrial transport. Significant alterations of fatty acid oxidation proteins were only observed in the mitochondrial patient’s iNPCs (Fig. 8A). VAPB and proteins acting downstream from VAPB were significantly down-regulated only in iNPCs of patient 1 (Fig. 8B). VAPB binds to protein tyrosine phosphatase interacting protein 51 (PTPIP51), and is known to be involved in connecting ER with mitochondria as part of the mitochondria-associated ER membranes (MAM) complex (14). Based on the differentially expressed proteins we identified several biological processes different in the mitochondrial or neuropathy cell lines (Supplementary Material, Table S1).

### Mitochondrial calcium uptake was significantly altered in fibroblasts and iNPCs of patients carrying pathogenic *GARS* mutations

**Figure 9.**
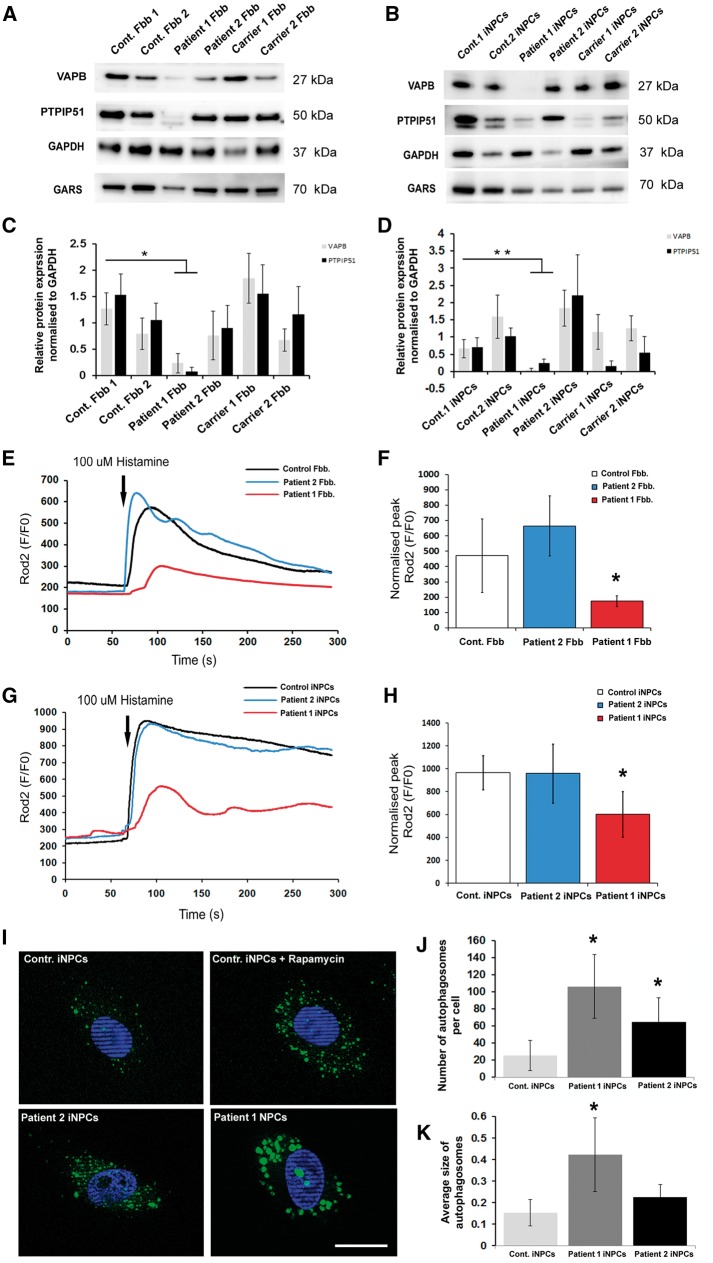
Western blot analysis and Ca^2+^ measurement in control and patient cell lines. (**A**, **B**). Total protein lysate from control and patient fibroblasts and iNPCs cells were analysed by immunoblotting using VAPB, PTPIP51, GAPDH and GARS antibodies. Representative blots from three independent experiments are shown. (**C**, **D**) Bars represent the average protein expression levels normalized to GAPDH obtained by densitometry analysis. Error bars show standard deviation, **P < 0.005, *P < 0.05. Data were analysed with paired *t*-test on Sigma plot (version 11.0) where samples represents *n* = 3 (experimental replicates). Mitochondrial Ca^2+^ transients in fibroblasts (**E**) and iNPCS cells (**G**) are shown. Mitochondrial Ca^2+^ transients were induced by cell stimulation with 100 μm histamine. The traces are representative of at least 10 independent experiments. *Bars* indicate the average heights of peak values (**F**, **H**). Results are the mean ± Standard deviation *P < 0.05. Data were analysed with unpaired *t*-test on Sigma plot (version 11.0) where samples represent *n* = 10 (experimental replicates). The *P*-values are from an unpaired *t*-test, *P < 0.05. Cell line of patient 1 harbouring the dominant *GARS* mutation increased the number and size of the autophagic structures. Representative images of cells are shown with DAPI-labelled nuclei. Scale bars are 10 um (**I**). Bar charts show quantification of autophagosomes (dots/cell) (**J**) and average size of the autophagic structures (**K**). *n* = 30–50 cells per condition in three independent experiments. Data were analysed by one-way ANOVA and Dunn’s method. Error bars are standard deviation *P < 0.05.

To confirm the altered expression of VAPB in patient cells we performed immunoblotting for VAPB and its interacting partner PTPIP51 in both fibroblasts and iNPC cells (Fig. 9A and B). Our data indicated significantly reduced levels of VAPB and PTPIP51 in the cells of patient 1, and more importantly the lack of VAPB protein was more evident in the iNPCs compared to fibroblasts (Fig. 9B).

The primary role of MAM is to facilitate delivery of Ca^2+^ to the mitochondrial matrix from ER stores. Thus, reduced levels of VAPB loosen the ER-mitochondrial associations and perturb this delivery. To address the possible role of GARS in mitochondrial Ca^2+^ homeostasis we monitored the mitochondrial Ca^2+^ uptake following its release, induced by histamine, from ER stores. For these experiments we used both fibroblasts and iNPCs cells. After recording the baseline where indicated (Fig. 9E and G), cells were exposed to 100 μM histamine (His), causing the generation of inositol 1, 4, 5 trisphosphate (InsP_3_) and the consequent opening of the InsP_3_ channels of the intracellular stores. Representative traces of typical experiments for fibroblasts and iNPCs are shown in Figure 9E and G. Mitochondrial Ca^2+^ influx in cells of patient 1 were significantly lower in both cell types than those observed in control or cells of patient 2. Peak values are shown in Figure 9F and H.

To complement these studies, we also analysed the autophagy in the iNPC cells. Recently published work showed that the VAPB-PTPIP51 tethers regulate autophagy by mediating the delivery of Ca^2+^ to the mitochondria from the ER stores (15). Autophagy detection kit was used specifically label autophagosomes (Fig. 9I). Fully in line with our findings we identified significantly enlarged and increased numbers of autophagic vacuoles in the neuropathy cell line compared with control cells (Fig. 9J and K).

### Investigations in the *Gars^C210R^* mice detected significant changes in MAM proteins and mitochondrial dysfunction in sciatic nerve

To investigate the effect of *GARS* mutations on mitochondrial translation and on MAM proteins we studied different tissues in *Gars^C201R^*mice. Homozygous *Gars^C201R/C201R^*mice have severe motor deficits and die between 14 and 17 days. Heterozygous *Gars^C201R/WT^*mice present with a reduction in axon diameters and muscle weakness at 3 months of age (16). First we studied mitochondrial protein synthesis by ^35^S methionine pulse-labelling in primary fibroblasts isolated from wild-type, heterozygous and homozygous mice. Our data, using Coomassie blue stained gel as loading control, showed normal result (Supplementary Material, Fig. S6). We could not detect a mitochondrial translation defect in mouse fibroblasts, similar to our data in patient fibroblasts.

**Figure 10.**
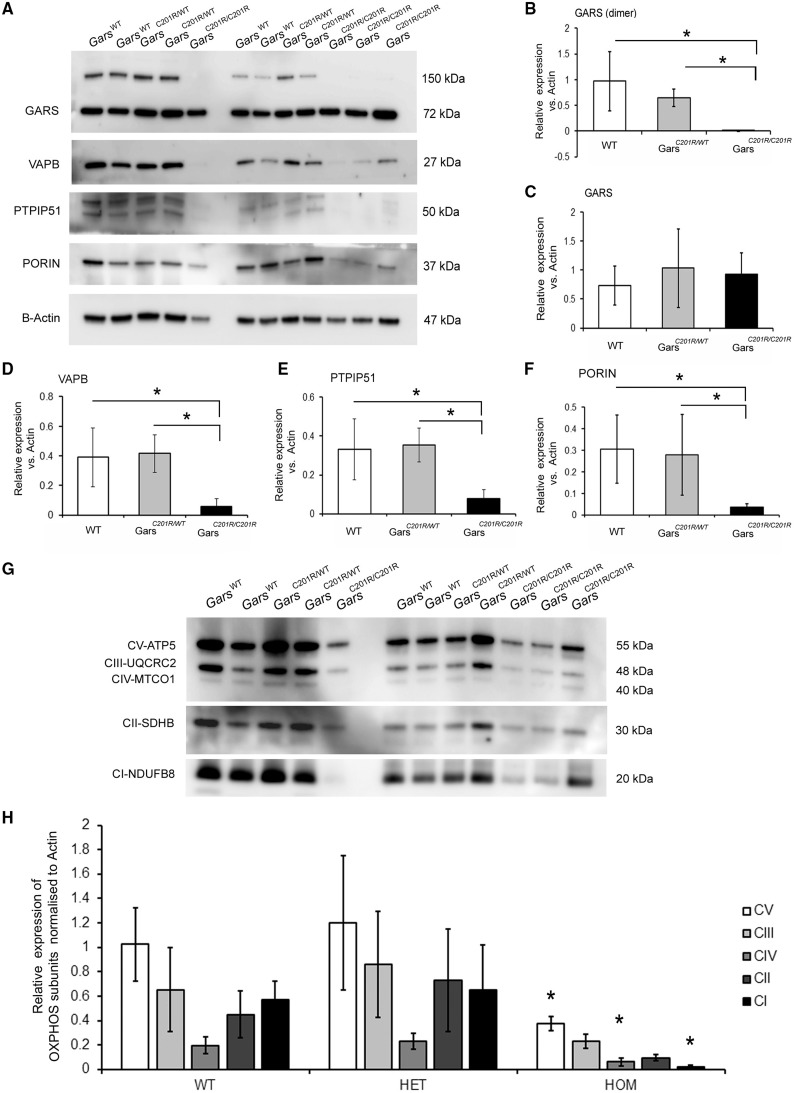
Investigations of mitochondrial function in different tissues of the *Gars^C210R^* mouse. (**A**) Western blot shows the level of MAM proteins and mitochondrial subunits in wild type, *Gars^C201R/wt^* and *Gasr^C201R/C201R^* mouse sciatic nerve. Sciatic nerves were isolated at P7 and equal amounts of protein were analysed. Actin was used as a loading control for total cell lysates. (**B**–**F**) Graphed data are presented as mean (± SD) from three separate experiments. Data were analysed with unpaired *t*-test on Sigma plot (version 11.0) where samples represents *n* = 6. (**G**) Immunoblotting for subunits of mitochondrial complexes. Total tissue lysate isolated from sciatic nerve of wild-type (WT), heterozygous (*Gars^C201R/WT^*) and homozygous (*Gars^C201R/C201R^*) animals at P7. Mouse tissues were incubated with different antibodies CI subunit (NDUFA9), CII subunit (SDHA), CIII (UQCRC2), CIV subunit (COX IV) and CV subunit (ATP5A). Actin was used a loading control. (**H**) Densitometry of western blotting was performed using ImageJ software. Data in arbitrary units (a.u.) represent the mean ± SD of three separate experiments (*n* = 6). Data were analysed with unpaired *t*-test on Sigma plot (version 11.0), *P < 0.05 compared with WT samples.

Next, we investigated the expression level of VAPB and PTPIP51 in isolated sciatic nerve at P7. We detected significantly reduced levels of VAPB and its interacting partner PTPIP51 in homozygous *Gars^C201R/C201R^*mice while no changes were found in heterozygous *Gars^C201R/WT^*mice (Fig. 10A, D and E). Furthermore, detection of GARS protein revealed a complete lack of gars dimers in *Gars^C201R/C201R^*animals which are the active form of the enzyme [Fig. 10A (upper band) and B]. On the other hand the level of GARS monomer was unaltered compared to wild-type littermates [Fig. 10A (lower band) and C].

Based on our human iNPCs data indicating mitochondrial defect in patients, we also studied the level of mitochondrial proteins in different mouse tissues. The amount of mitochondria detected by porin expression suggested significant decrease (Fig. 10A and F) in the sciatic nerve of the *Gars^C201R/C201R^*animals and we detected significantly lower level of complex I, IV and V subunits (Fig. 10G and H). Other highly metabolic tissues were also analysed (skeletal muscle, brain, heart, liver and kidney) at P0 and P14. The expression level of mitochondrial respiratory chain complexes were normal in all mice (Supplementary Material, Figs S4A–D and S5A–D) at both time points, and only a mild, not significant decrease of complex I was detected in skeletal muscle of both heterozygous and homozygous mice (Supplementary Material, Fig. S5E) at P14. Measurements of enzyme activities also showed normal levels in these tissues (Supplementary Material, Figs S1F–I and S2F–I).

In summary, extensive investigations in the *Gars^C210R^* mice detected tissue-specific mitochondrial dysfunction and altered level of MAM proteins in sciatic nerve only, while all other tissues showed normal results.

## Discussion

It has been shown in mice that mutant GARS acquires a neomorphic binding activity to neuropilin 1 (Nrp1) receptors that directly antagonizes an essential signalling pathway for motor neuron survival (9). This aberrant interaction interferes with the binding of the vascular endothelial growth factor (VEGF) to Nrp1. Genetic reduction of Nrp1 in mice worsens neuropathy, whereas enhanced expression of VEGF improves motor function. It seems to be an important pathomechanism of the secreted GARS outside the neurons however it does not explain all abnormalities observed in *GARS* mutations.

In this study we identified that reduction or loss of GARS (by siRNA down-regulation or some pathogenic mutations) result in decreased translation of mtDNA and nuclear encoded subunits confirming that GARS is important for obtaining mitochondrial function, and that loss of function of GARS result is reduced mitochondrial translation. We showed that siRNA down-regulation of GARS resulted in reduced mitochondrial translation in neuronal cells and myoblasts, but not in fibroblasts, suggesting that *GARS* mutations may lead to a tissue-specific mitochondrial translation defect due to loss of function. We could not demonstrate a defect of mitochondrial protein synthesis in a mouse model of GARS (Gars^C201R^) in five different tissues. The steady-state level of Gars was not altered by the mutation, and we detected redistribution of Gars into mitochondria, which may have prevented respiratory chain dysfunction, as higher protein levels of Gars were detected in mitochondria in the studied tissues.

Furthermore, we detected complex alterations of mitochondrial metabolism, which cannot be fully explained by a defect of cytosolic and mitochondrial translation suggesting that GARS has other non-canonical functions within neurons (17,18). The canonical role of GARS would be the addition of glycine residues to the cognate tRNA during mitochondrial and cytosolic protein translation and we focussed on studying mitochondrial translation. We detected partial co-localization of GARS with mitochondrial RNA granules. Mitochondrial RNA granules are the centres of posttranscriptional RNA processing and the biogenesis of mitochondrial ribosomes (18) and provide a platform for the spatiotemporal regulation of numerous processes including RNA maturation, ribosome assembly and translation initiation (19), supporting the role of GARS in mitochondrial translation and raising the possibility that mutant GARS may cause alteration of these functions. However mitochondrial RNA granules are not specific for neurons and do not explain the tissue specificity in the pathomechanism of *GARS* mutations.

We generated iNPCs by direct conversion from human fibroblasts and show that this is an excellent cellular model to study tissue-specific mechanisms in neurons. We detected an abnormal translation of mtDNA and nuclear-encoded mitochondrial proteins and abnormal mitochondrial respiration in neuronal cells, but not in fibroblasts of patients compared with controls. Interestingly, these changes were more prominent in iNPCs of the dHMN-V patient carrying a dominant, probably gain-of-function *GARS* mutation (patient 1), suggesting that some neuropathy-associated mutations result in more severe or complex alteration of mitochondrial function in neurons.

We further investigated the mitochondrial defect by RNAseq and by comparative proteomics in iNPCs carrying autosomal dominant and recessive *GARS* mutations which showed several significant changes of the mitochondrial transcriptome and proteome. Altered levels of mitochondrial respiratory chain subunits, Krebs cycle enzymes and proteins involved in mitochondrial transport and respiratory chain assembly were detected in both mutant iNPCs. In addition, significant changes were identified in the fatty acid metabolism only in iNPCs with recessive *GARS* mutations (Fig. 8A). In support of the relevance of these findings, exercise intolerance, myopathy and cardiomyopathy, typically present in defects of fatty acid oxidation, were observed in the patients reported to date with recessive *GARS* mutations (20).

iNPCs carrying a neuropathy-related dominant *GARS* mutation showed reduced VAPB and its downstream pathways, including altered mitochondrial and cellular calcium homeostasis, providing a potential explanation for neuron-specific clinical manifestations (Fig. 8B). Autosomal dominant mutations in *VAPB* were reported in several families world-wide with clinical presentations of ALS (ALS8) or distal spinal muscular atrophy (14,21). VAPB is part of the mitochondria-associated ER membrane (MAM) complex and was shown to bind to the outer mitochondrial membrane protein PTPIP51 to tether ER to mitochondria and plays an important role in the quality control and provides an additional level of flexibility of neurons to cope with misfolded protein stress in the ER (22). Modulating VAPB induces changes in ER-mitochondria contacts and Ca^2+^ exchange between the two organelles, and predicted to lower mitochondrial ATP production (22). It has been very recently shown that the VAPB-PTPIP51 tethers regulate autophagy through mediating delivery of Ca2+ to mitochondria from ER stores, providing a new molecular mechanism for regulating autophagy (15). In support of the relevance of reduced VAPB, we also detected low protein levels of its direct interacting partner PTPIP51 and several proteins involved in pathways downstream of VAPB were altered including calcium metabolism, ubiquitination, ephrin signalling, ER trafficking and vesicle fusion (SNARE) (Fig. 8B).

In support of the functional effect of reduced VAPB, we detected a significant defect of mitochondrial calcium metabolism in fibroblasts and neuronal cells in the dHMN-V patient (patient 1). Furthermore, significant increase of autophagy was observed in the patient cells (Fig. 9I and J) and the average size of autophagic structures was significantly increased specifically in patient 1 (Fig. 9K). Furthermore, extensive investigations in the *Gars^C210R^* mice detected tissue-specific mitochondrial dysfunction and altered level of MAM proteins in sciatic nerve only, while all other tissues showed normal results. Based on the reduced level of MAM proteins and respiratory chain subunits we think that the homozygous p.Cys201Arg mutation has a loss-of-function effect in *Gars^C201R^* mice, while we cannot provide experimental evidence of the loss of function in the heterozygous animals.

Although further studies are needed to explore the exact mechanism of the GARS/VAPB connection we suggest that mutant GARS has an impact on mitochondrial calcium metabolism and ER–mitochondria interactions which contribute to the neuron-specific clinical presentations. Furthermore, the changes detected in our study may provide a functional link between the previously identified presynaptic defects of neuromuscular transmission and *GARS* mutations, as calcium signalling is important for vesicle refinement at the neuromuscular junction (23).

The importance of MAM in motor neurons has been highlighted by the fact, that mitofusin 2 (MFN2), a common cause of axonal CMT (CMT2), has been suggested to act as an ER-mitochondria tether, and its ablation decreases interorganellar communication (24). Many functions regulated by ER–mitochondria associations have been shown to contribute to neurodegenerative diseases such as amyotrophic lateral sclerosis/frontotemporal dementia (ALS/FTD), Alzheimer’s disease (AD) and Parkinson’s disease (PD), however the exact mechanism involved in damage of different neuronal cell types in these diseases needs further investigations. Here we show that the ER-mitochondria axis is altered in GARS-related neuropathy, highlighting that MAM is particularly important in motor neurons.

In summary, we show that a different type of mitochondrial dysfunction underlies autosomal recessive and dominant *GARS* mutations. While a defect of mitochondrial protein synthesis in recessive GARS-related mitochondrial disease is most likely a result of loss of function, in dominant neuropathy-causing mutations we identified a defect of the MAM complex, suggesting that mitochondria ER interactions and calcium metabolism, possible due to non-cognate function of GARS may potentially explain tissue-specific clinical manifestations in human motor neurons.

## Materials and Methods

### Cell culture

Cultured myoblasts were grown in skeletal muscle cell growth medium and supplement mix (PromoCell) supplemented with 10% (v/v) foetal bovine serum (FBS, Sigma Aldrich) and 4-mM L-glutamine (Invitrogen) and cultured as recommended by the supplier. Fibroblasts were grown in high glucose Dulbecco’s modified Eagle’s medium (DMEM, Sigma, Poole, UK) supplemented with 10% FBS. Myoblasts cell line was immortalized by transduction with a retroviral vector expressing the catalytic component of human telomerase (25). Human oligodendrocyte cells (MO3.13) and a human bone marrow neuroblastoma cell line (SHSY-5Y) were grown in DMEM: F12 supplemented with 10% FBS. The human oligodendroglia cell line was a kind gift of Dr. Josephine Nalbantoglu from the McGill University, where it was originally characterized.

### Conversion of skin fibroblasts to iNPCs

The method used was described previously in Meyer *et al.* (11).

### siRNA-mediated down-regulation

Silencer GARS siRNA (number siRNA, Ambion–Life Technologies) was transiently transfected in control myoblasts, fibroblasts and in SHSY-5Y human neuroblastoma cell line at a final concentration of 20 nM using Lipofectamine RNAiMAX (Invitrogen), according to the manufacturer’s specifications. Transfections were repeated on day 3 and day 6, cells were harvested on day 9. A non-targeting *Silencer*Select Negative Control (#1) was used as a control.

### BN PAGE, in-gel activity and immunoblotting

About 3% and 15% gradient gels and a 4% stacking gel were prepared for BN PAGE according to the protocol of Calvaruso *et al.* (26). A Gilson MiniPuls 3 gradient gel mixer (Gilson, USA) was used at a speed of 5.38 ml/min. SBG buffer [750 mM aminocaproic acid, 5% Coomassie Brilliant Blue G250 (Biorad, UK)) was added to the samples and 2 μg of each sample used. Following electrophoresis the gel was transferred to a PVDF membrane, de-stained, blocked and incubated with primary antibody to the respiratory chain complexes (complex I subunit NDUFA9—2 μg/ml, complex II 70 kDa subunit—0.2 μg/ml, complex III core 1 subunit—1 μg/ml, complex IV subunit 4–1 μg/ml, complex V ATP synthase—0.25 μg/ml, all Abcam, UK), a secondary rabbit anti-mouse antibody (Dako, UK) (0.5 μl per ml) and then developed with ThermoScientific Pierce ECL2 Western Blotting kit or BioRad Clarity Western ECL Solution. The signal was detected with the UVP BioSpectrum 500 Imaging System and the intensity quantified using Image J software. ‘In-gel’ assays were carried out as described (27).

### Pulse-labelling of mitochondrial translation products

*In vivo* ^35^S-metabolic labelling studies were performed as described previously with the following modifications. Cells, cultured to 60–70% confluency in T25 mm flasks, washed with phosphate-buffered saline (PBS; Sigma) and washed by incubating twice for 10 min at 37°C/5% CO_2_ in methionine/cysteine-free DMEM (Sigma, Poole, UK), with the media replaced between each incubation. Cells were then incubated for 15 min at 37°C/5% CO_2_ in methionine/cysteine-free DMEM supplemented with 5% (v/v) dialyzed FBS, 0.1 mg/ml emetine dihydrochloride (Sigma). Following addition of 200 mCi/ml ^35^S-methionine/cysteine (^35^S EasyTag EXPRESS; Perkin Elmer), cells were incubated for 15 min at 37°C/5% CO_2_, then washed twice with ice-cold DMEM supplemented with 7.5 mg/ml methionine. Cell pellets were prepared after washing once with ice-cold PBS. Radio-labelled proteins were then analysed using SDS–PAGE as described previously.

### Mitochondrial extraction from mouse tissues

Unless specified all chemicals were purchased from Sigma (Sigma Aldrich, UK). Liver, brain kidney heart and muscle tissue was weighed and added to a glass Elvehjem potter with 10 volumes of Buffer A (0.32 M sucrose, 10 mM Tris–HCl and 1 mM EDTA) to the weight of tissue. The homogenate was then centrifuged at 1000*g* for 5 min. The remaining supernatant was centrifuged at 9000*g* for 10 min and the pellet re-suspended in 100 μl Buffer A with digitonin (1 in 200 of weight of tissue). Protease inhibitor tablet (Sigma) in 10 ml PBS (PI/PBS) was added to dilute the digitonin and the sample was centrifuged at 10 000*g* for 10 min. The pellet was re-suspended in 30–100 μl of MB2 buffer [0.5 ml 3× gel buffer (1.5 M aminocaproic acid, 150 mM Bis–Tris, pH 7), 0.5 ml 2 M aminocaproic acid, 4 μl 500 mM EDTA) depending on its size. N-dodecyl *B*-d-maltoside in PI/PBS was added to a final concentration of 1%, vortexed, incubated on ice for 15 min and then centrifuged at 16 000*g* for 30 min. The supernatant was removed and saved.

### Gene expression studies

cDNA was generated by reverse transcription of 500 ng of total RNA using the Superscript VILO cDNA synthesis kit (Invitrogen, 11754-050) according to manufacturers’ instructions. qPCR (Applied Biosystems 7900HT) was performed in triplicate on cDNA using SYBR Green PCR Master Mix (Invitrogen, 4309155). Samples were normalized using the average of two reference genes, GAPDH and actin. Primers are shown in Supplementary Material, Table S2.

### Immunofluorescence

Cells were fixed with 4% paraformaldehyde for 15 min and washed 3× with Tris-buffered saline (TBS) before the blocking solution consisting of TBS with 10% donkey serum, 0.1% Triton X-100 and 0.1% Tween-20 was applied for 1 h. All primary antibodies were diluted in blocking solution (MTCO2, Abcam; MTCO1, Abcam; NDUFS8, Abcam; NDUFS2, Abcam; GARS, Proteintech; S-100Beta, Abcam; Collagen 1, Abcam; Nestin, Milliopore; GRFS1, Abcam; eIF4E, Abcam; Mitotracker, Invitrogen). Incubation of the primary antibody was performed overnight at 4°C. The next day, cells were washed 3× in TBS before the secondary antibody (Alexa-Fluor Anti-Rabbit 488, Alexa-Fluor Anti-mouse 594, Invitrogen) and DAPI diluted in blocking solution was applied for 1.5 h at room temperature. Immunofluorescence images were collected using a Zeiss Axio Imager Z1 fluorescence microscope equipped with Zeiss Apotome 2 (Zeiss, Germany) in AxioVisionRel 4.9 software.

### Oxygen consumption measurement

Oxygen consumption was measured in adherent fibroblasts and iNPCs with a XF96 Extracellular Flux Analyzer (Seahorse Bioscience Billerica, MA, USA) as described earlier (28). Each cell line was seeded in 12 wells of a XF96-well cell culture microplate (Seahorse Bioscience) at a density 30 × 10^3^ cells/well (20 × 10^3^ cells/well for iNPCs) in 80 ul of DMEM and incubated for 24 h at 37°C in 5% CO_2_ atmosphere. The cells were sequentially treated with oligomycin (1 μM) *p*-trifluoromethoxy carbonyl cyanide phenyl hydrazine (0.5 uM) antimycinA (1 μM) and rotenone (1 μM). Oxygen consumption rate (OCR), leaking respiration (LR), maximal capacity respiration (MCR) and not electron transport chain respiration (NMR) were determined by adding 1 μM oligomycin (Sigma-Aldrich, Dorset UK) (LR), carbonyl cyanide-*p*-trifluoromethoxyphenylhydrazone (FCCP) (Sigma-Aldrich, Dorset UK) (MCR: 2 injections of 0.5 μM and 1 μM respectively) and 1 μM Rotenone/antimycin (Sigma-Aldrich, Dorset UK) (NMR), respectively. The data were corrected by the NMR and expressed as pmol of oxygen/min/mg of protein. The quantity of protein was measured by Bradford assay (29).

### RNAseq analysis

Total RNA was isolated from control fibroblast cells and from patient and controls iNPC cells using the mirVana™ miRNA Isolation Kit (Ambion) and DNAse treated with the DNA-free™ DNA Removal Kit (Ambion). RNAseq libraries were prepared using Illumina (Illumina, Inc. California, USA) TruSeq Stranded Total RNA with Ribo-Zero Human kit and were sequenced on an Illumina HiSeq 2500 platform using paired-end protocol. The quality of sequencing reads was checked with FastQC (http://www.bioinformatics.babraham.ac.uk/projects/fastqc/; date last accessed February 20, 2018). Reads were aligned using the STAR (v2.5.2b) aligner and the 2-pass protocol that is outlined in the GATK documentation for calling variants from RNAseq (https://software.broadinstitute.org/gatk/; date last accessed February 20, 2018). Number of reads mapped to genes were counted using HTSeq-count (30). Differentially expressed genes were identified with Bionconductor package DESeq2 (31), comparing control fibroblast versus iNPC cells, as well as patient iNPC versus control iNPC cells. Genes differentially expressed with a false discovery rate (FDR) of ≤ 0.1 and a log 2 fold change ≥ 1 were considered as differentially expressed genes. Gene set enrichment analysis for gene ontology terms was performed using the CPDB web tool (http://cpdb.molgen.mpg.de/; date last accessed February 20, 2018).

### Proteomic analysis: sample preparation

Samples (two biological replicates per condition) were solubilized in 1% SDS (w/v), 50 mM Tris–Cl pH 7.8, 150 mM NaCl, supplemented with protease inhibitor Complete Mini and phosphatase inhibitor cocktail PhosSTOP (Roche, Switzerland). The Bichinonic acid assay (BCA) (Thermo Scientific, USA) was used to determine protein concentrations. Cysteines were reduced with 10 mM dithiothreitol for 30 min at 56°C and alkylated with 30 mM iodoacetamide for 20 min at room temperature, in the dark. Per sample, a volume corresponding to 10 µg of protein was 10-fold diluted with ice-cold ethanol, followed by 60 min of incubation at −40°C. The sample was centrifuged for 30 min at 4°C and 18 000*g* and the supernatant was discarded. The protein pellet was resolubilized in 5 µl of 4 M guanidinium chloride and diluted 20-fold with 50 mM ammonium bicarbonate (ABC). Trypsin (Promega, USA) was added in a ratio 1: 20 (trypsin: protein, w/w) and samples were digested at 37°C for 14 h. Digestion was stopped by adding trifluoroacetic acid to a final concentration of 1%. Digestions were quality controlled by separation of 1 µg on a monolithic column according to Burkhart *et al.* (32).

### Nano-LC-MS/MS analysis

Per sample 0.5 µg were analyzed using an Ultimate 3000 nano RSLC (Thermo Scientific) coupled to an Orbitrap Fusion Lumos (Thermo Scientific, Germany). Peptides were pre-concentrated on a 75 μm × 2 cm C18 Acclaim Pepmap trapping column (Thermo Scientific, Germering, Germany) in 0.1% TFA for 10 min using a flow rate of 20 µl/min, followed by separation on a 75 μm × 50 cm Acclaim Pepmap C18 main column using a binary gradient (A: 0.1% formic acid, B: 84% acetonitrile, 0.1% formic acid) ranging from 3% to 30% B in 85 min at a flow rate of 250 nl/min. Samples were analysed in data-dependant acquisition mode (DDA). MS survey scans were acquired in the Orbitrap from 300 to 1500 *m*/z at a resolution of 120 000 using the polysiloxane ion at 371.1012 m/*z* as lock mass (33). For MS/MS precursors were selected using the top speed option (3 s) and a dynamic exclusion of 15 s. Peptides were selected with an isolation window of 1.2 *m*/*z* and fragmented using higher-energy collisional dissociation (HCD) with a normalized collision energy of 30%, and fragment ion spectra were acquired in the ion trap in rapid mode. Automatic gain control (AGC) settings were 2 × 10^5^ (MS) and 2 × 10^3^ (MS/MS) ions, maximum injection times were set to 50 ms (MS) and 300 ms (MS/MS), respectively (34,35).

### Mitochondrial calcium metabolism studies

Fibroblast and iNPC cell lines were seeded (100 000 cells/dish) on glass bottom dishes and allowed to adhere overnight. Rhod-2-am (ThermoFisher) was used to evaluate mitochondrial Ca2+, respectively. Rhod-2 (8 μM) was loaded at room temperature for 30 min. Cells were washed twice with PBS and incubated in tyrodes solution [137 mM NaCl, 2.7 mM KCl, 1 mM MgCl_2_, 0.2 mM Na_2_HPO_4_, 12 mm NaHCO_3_, 2 mM CaCl_2_, 10 mM Glucose, pH 7 (Sigma-Aldrich) for 15 min]. Dynamic measurements of mitochondrial Ca2+ were performed at room temperature with a Nikon A1R inverted confocal microscope equipped with a ×60, 1.40 NA oil objective in resonant scanning mode at 3.7 fps for 5 min. After 1 min (providing basal fluorescence) histamine (100 μM; Sigma-Aldrich) was added to evoke IP3R-dependent ER Ca2+ release and the Ca2+ transient was monitored. Image analysis was performed in Fiji, the background was subtracted and Ca2+ levels were calculated as (*F* – *F*0)/*F*0 where *F* indicates fluorescence and *F*0 basal fluorescence.

### Autophagy detection

Control and patient fibroblasts and iNPCs were cultured on coverslips and subjected to autophagy activity assays as described in the manufacturer instructions (Autophagy Detection Kit ab139484, Abcam, Cambridge, MA). For positive control cells were subjected to Rapamycin for 14–16 h.

### Proteomic data analysis

Data analysis for label-free quantification was performed with the Progenesis QI from Nonlinear Dynamics (Newcastle upon Tyne, UK). MS raw files were automatically aligned, followed by peak picking. Only peptides with charge states +2, +3 and +4 were considered for peptide statistics, analysis of variance (ANOVA) and principal component analysis (PCA). Only MS/MS spectra with ranks 1–10 were exported as peak lists with a limited fragment ion count 100. Using searchGUI (33) spectra were searched against a target/decoy version of the human Uniprot database (downloaded July 2015, containing 20 207 target sequences) using X! Tandem Vengeance (2015.12.15.2). Additionally, spectra were searched using Mascot 2.5 (Matrix Science). Search parameters were: trypsin as enzyme with a maximum of two missed cleavages, carbamidomethylation of Cys set as fixed modification, oxidation of Met set as variable modification, MS and MS/MS tolerances of 10 ppm and 0.5 Da, respectively. PeptideShaker 1.10.3 was used to combine all search result files and filter data at a FDR of 1% on the protein and peptide level (34). Obtained identifications were exported using the advanced PeptideShaker features that allow direct re-import of the quality-controlled data into Progenesis.

Only proteins quantified with at least one unique peptide were exported from Progenesis. Then, for each protein, the average of the normalized abundances (obtained from Progenesis) from the biological replicate analyses was calculated to determine the ratios between the samples and control. Only proteins which were (i) commonly quantified in all the replicates with (ii) at least two unique peptides, (iii) an ANOVA *P*-value of <0.05 (Progenesis) and (iv) a 2-fold regulation between samples and controls were considered as regulated.

A set of 85 regulated proteins were used to generate a high confidence STRING network (http://string-db.org/) and to analyse the enrichment of Gene Ontology terms.

## Supplementary Material 

Supplementary Material is available at *HMG* online.

## Supplementary Material

Supplementary Data
