# Supplementary material for: Mutations in glycyl-tRNA synthetase impair mitochondrial metabolism in neurons
Source: Hum Mol Genet. 2018 Apr 10;27(12):2187–204. doi: 10.1093/hmg/ddy127 (PMC5985729; doi:10.1093/hmg/ddy127)
Supplement: Supplementary Data [file ddy127_supplementary_data.doc]

**Supplementary data**

**Supplementary Figure 1.**


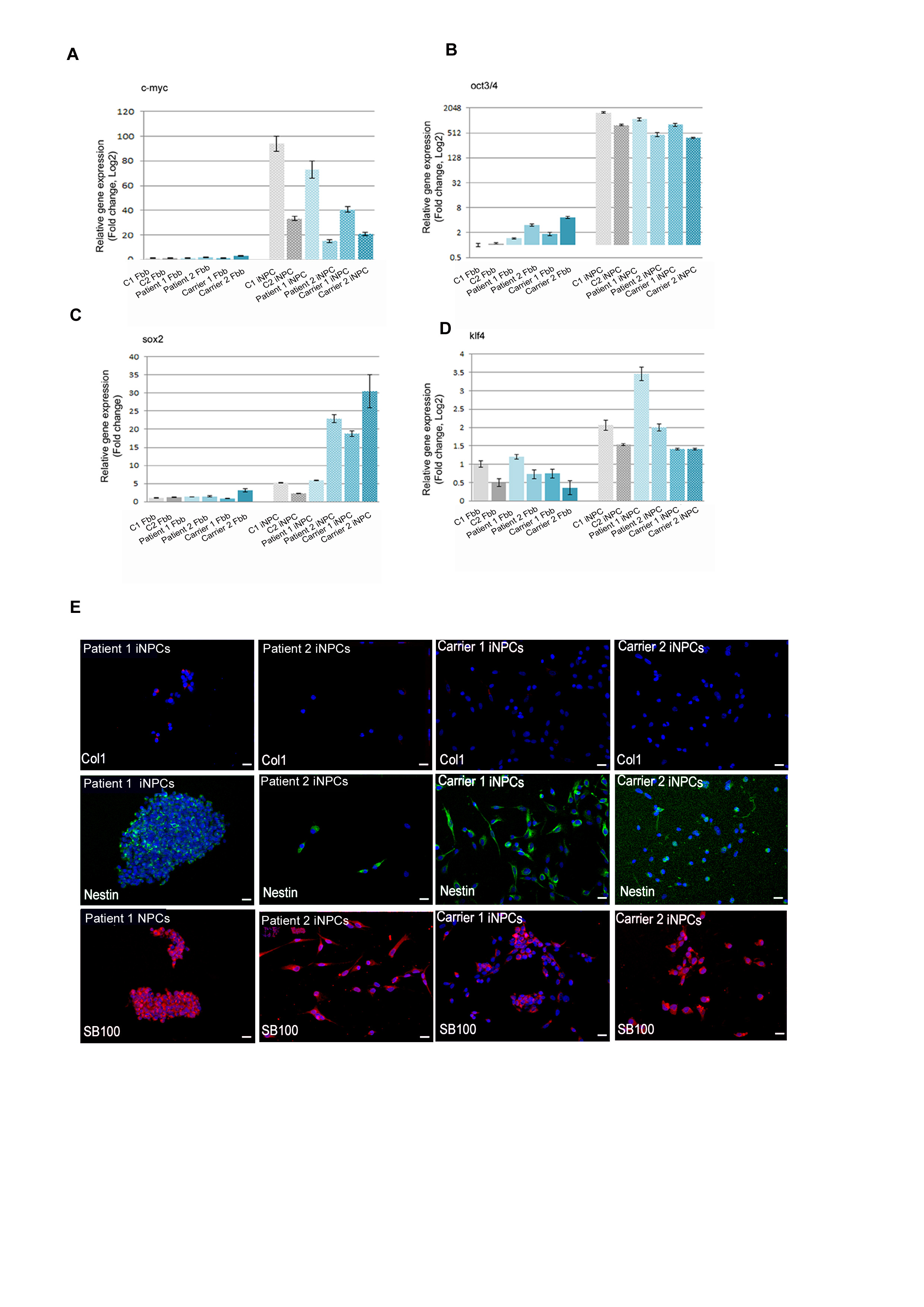


**Supplementary Figure 2.**


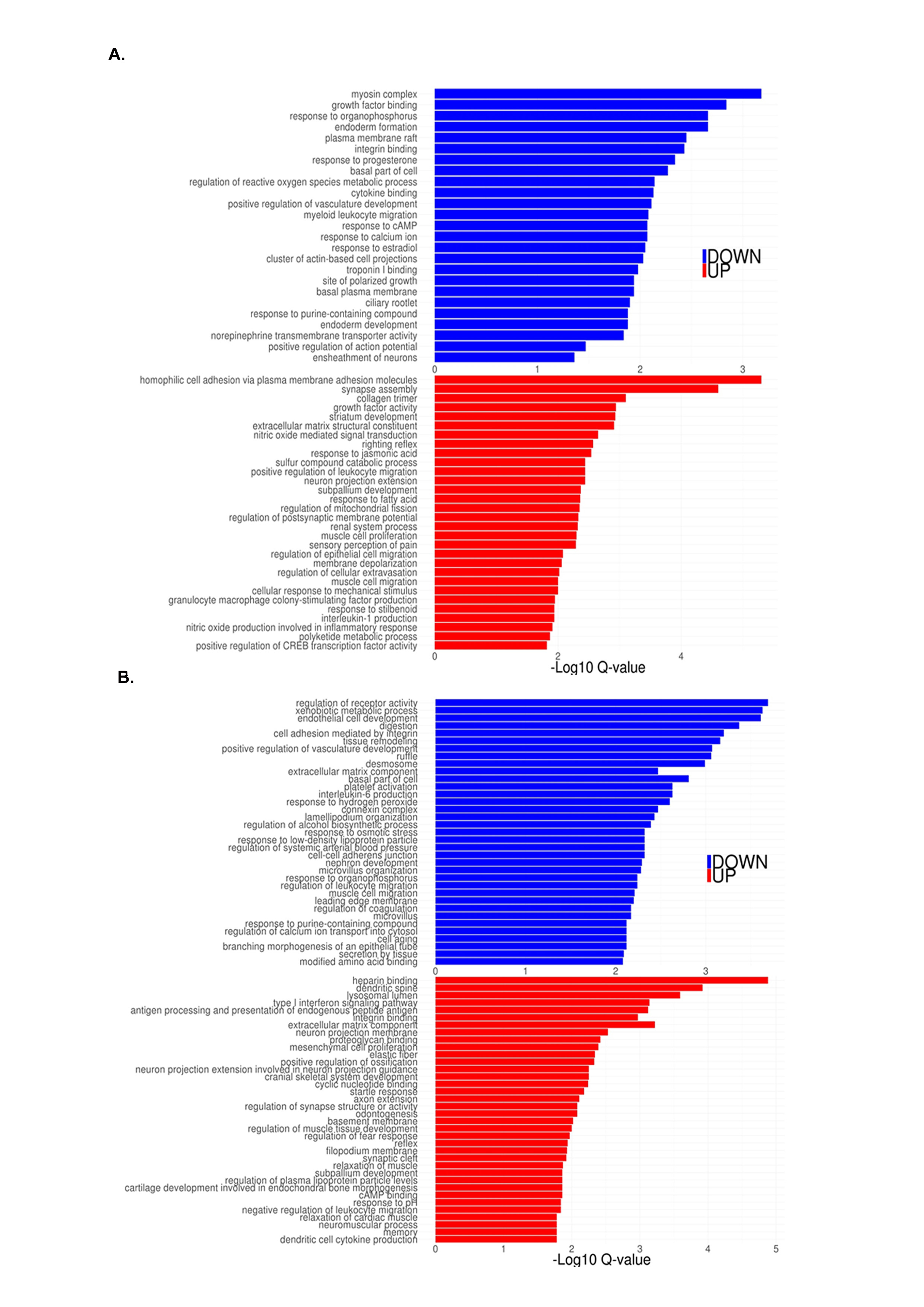


**Supplementary Figure 3**


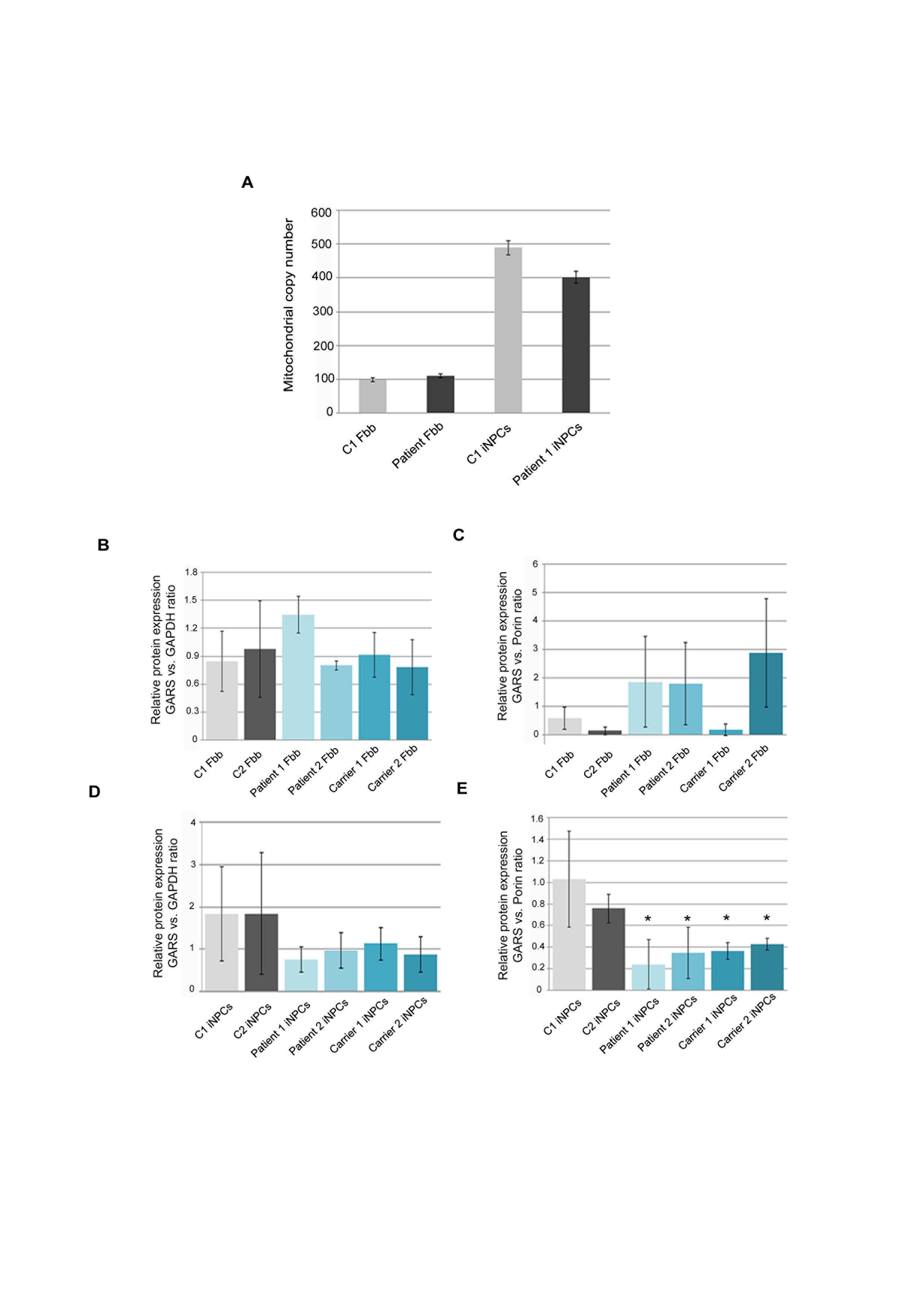


**Supplementary Figure 4.**


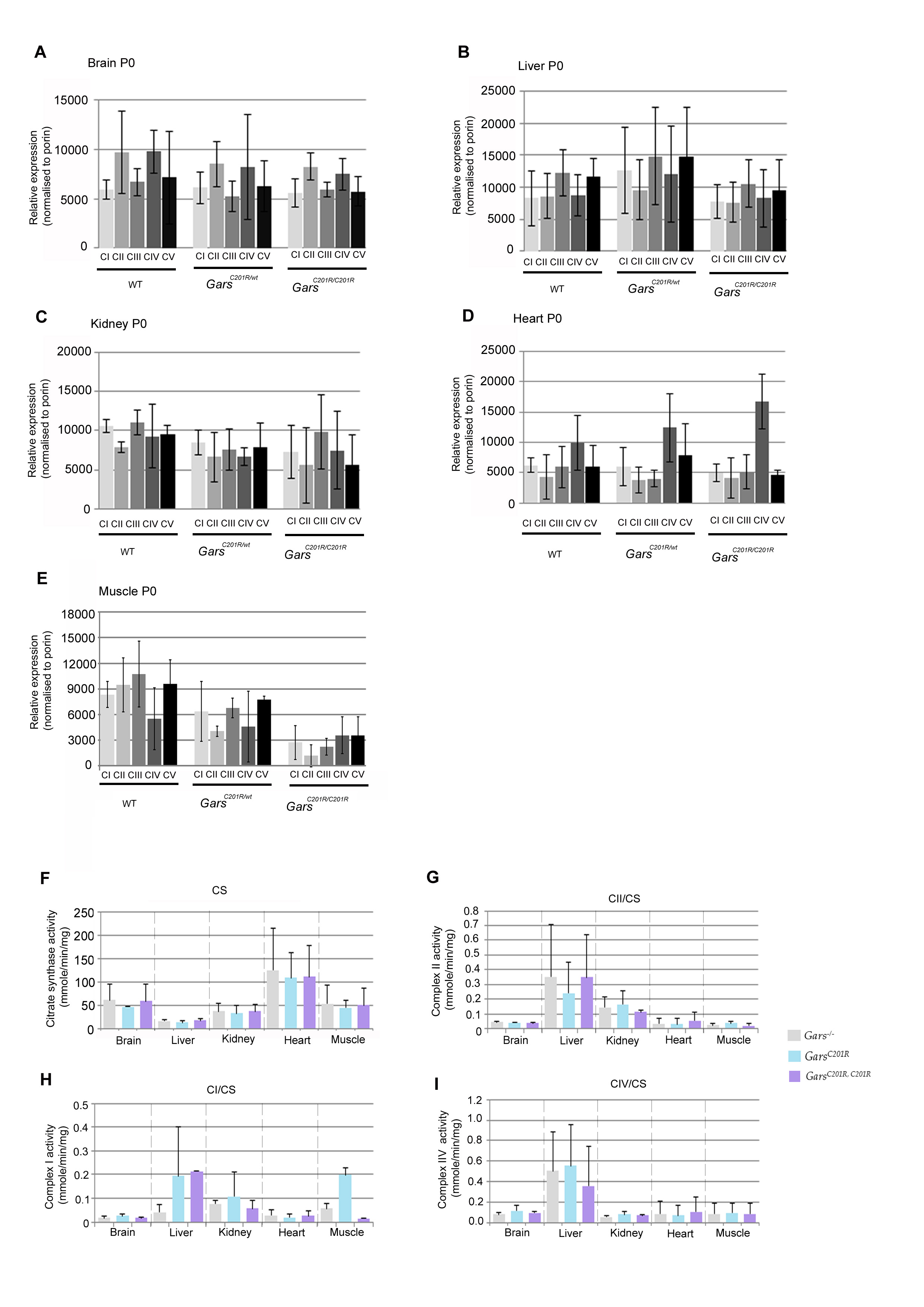


**Supplementary Figure 5.**


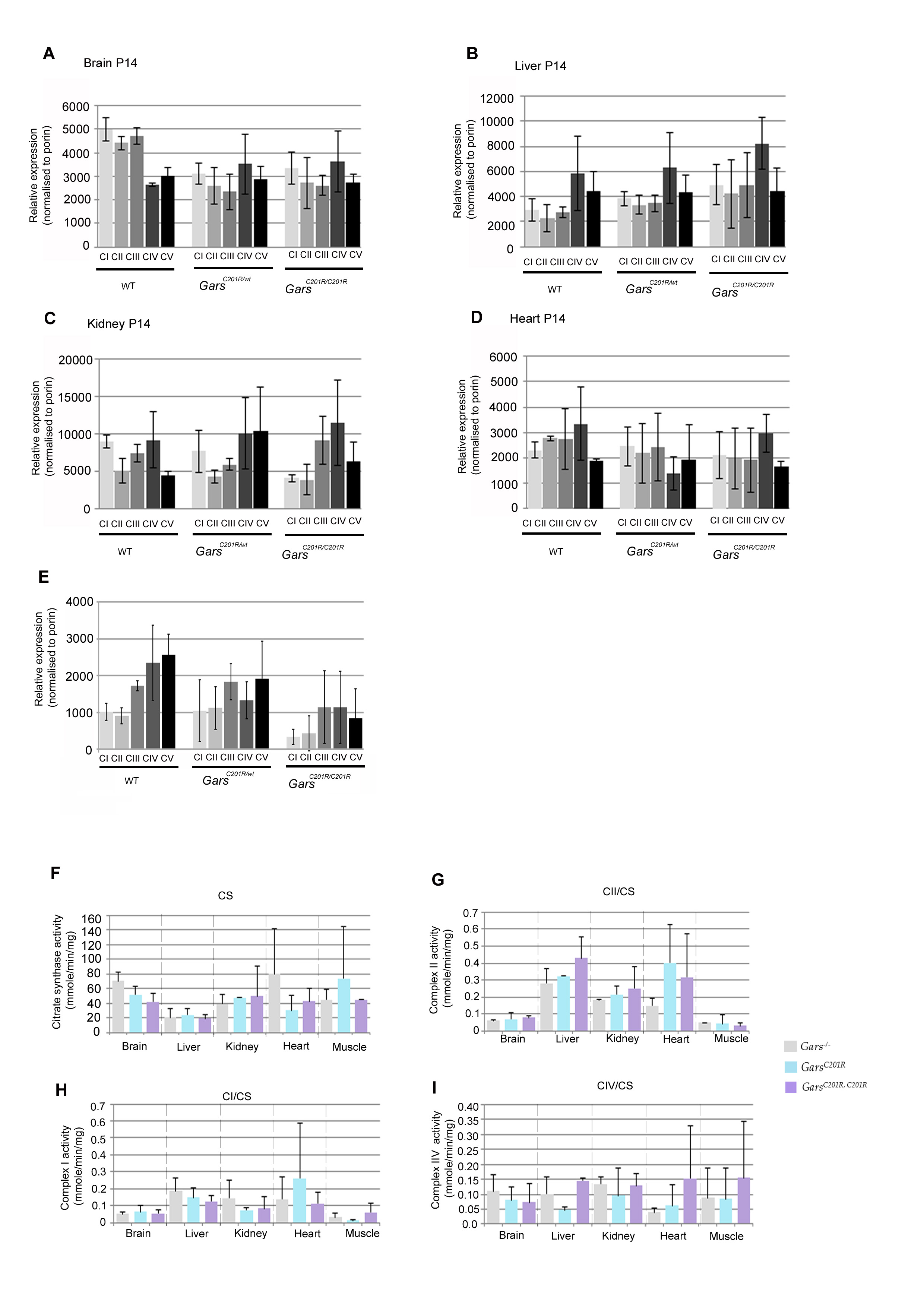


**Supplementary Figure 6.**


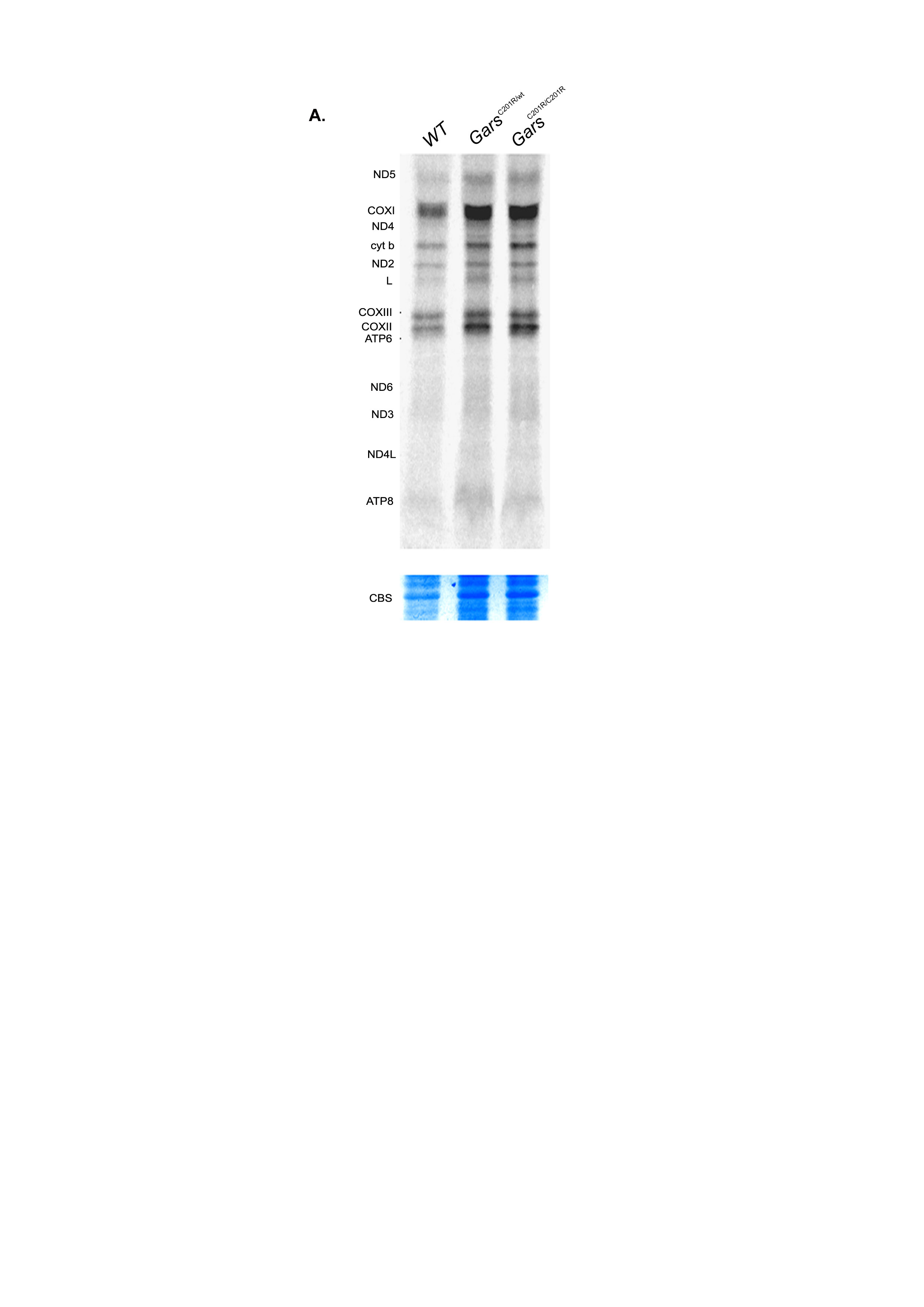


**Supplementary Table 1.**


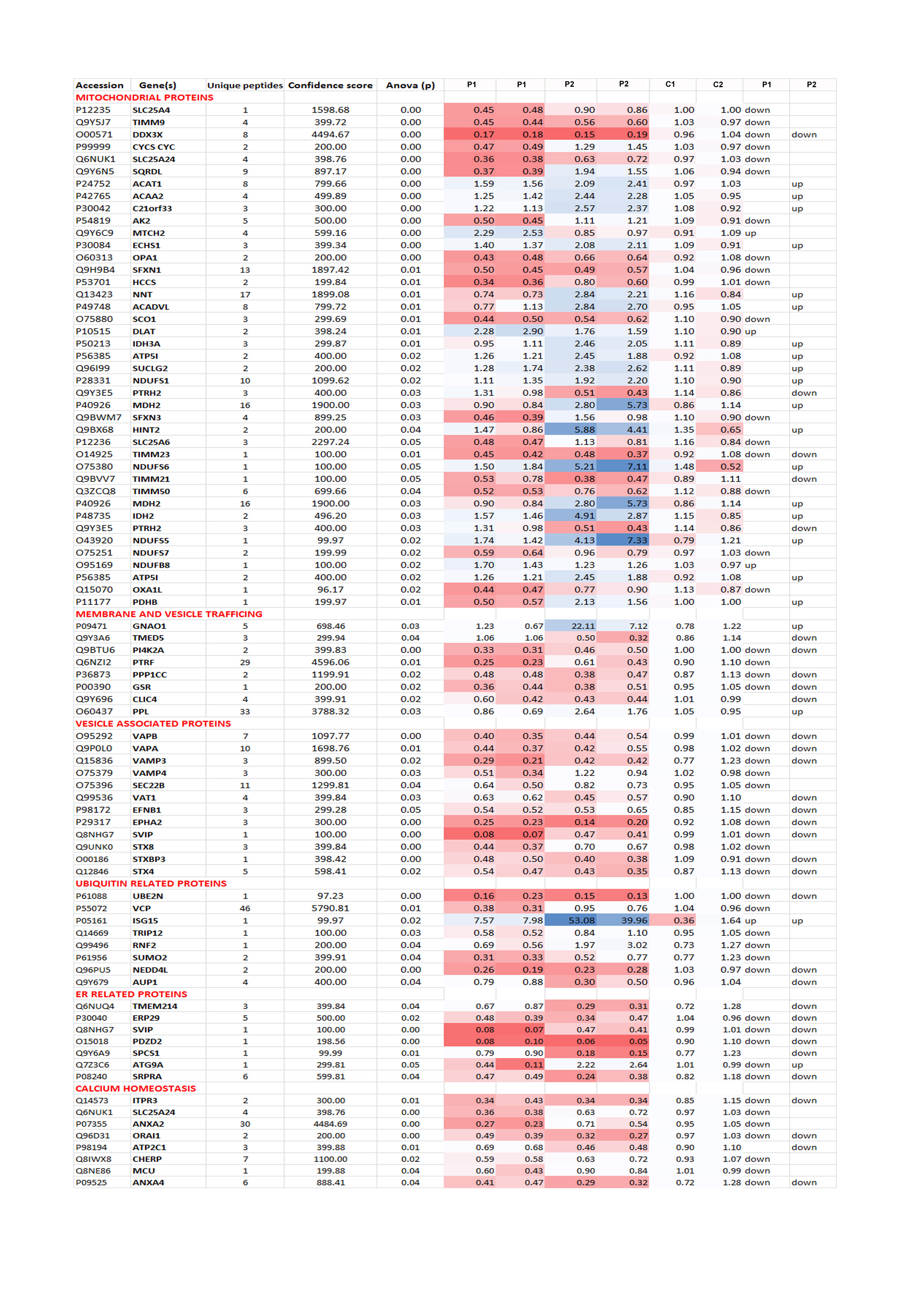


**Supplementary Table 2.**

| Nestin Fw | TCAAGATGTCCCTCAGCCTGGA |
| --- | --- |
| Nestin Rev | AAGCTGAGGGAAGTCTTGGAGC |
|  |  |
| Sox2 Fw | GCTACAGCATGATGCAGGACCA |
| Sox2 Rev | TCTGCGAGCTGGTCATGGAGTT |
|  |  |
| Snail1 Fw | TGCCCTCAAGATGCACATCCGA |
| Snail1 Rev | GGGACAGGAGAAGGGCTTCTC |
|  |  |
| Col1a1 Fw | GATTCCCTGGACCTAAAGGTGC |
| Col1a1 Rev | AGCCTCTCCATCTTTGCCAGCA |
|  |  |
| GAPDH Fw. | GTCTCCTCTGACTTCAACAGCG |
| GAPDH Rev. | ACCACCCTGTTGCTGTAGCCAA |
|  |  |
| Actin Fw | AGAGCTACGAGCTGCCTGAC |
| Actin Rev | AGCACTGTGTTGGCGTACAG |
|  |  |
| Ncan Fw | CCAATGAGGTGGATATGCCTGG |
| Ncan Rev | TCGGCTTTCTCTAGCTCCAGGA |
|  |  |
| C-myc Fw | CCTGGTGCTCCATGAGGAGAC |
| C-myc Rev | CAGACTCTGACCTTTTGCCAGG |
|  |  |
| Klf4 Fw | CATCTCAAGGCACACCTGCGAA |
| Klf4 Rev | TCGGTCGCATTTTTGGCACTGG |
|  |  |
| Twist 2 Fw | CTTATGTTTGGGGGGAGGTT |
| Twist 2 Rev | TAGCCAAGCAATCACGGAGA |
|  |  |
| DKK Fw | ATTCCAACGCTATCAAGAACC |
| DKK Rev | CCAAGGTGCTATGATCATTACC |
|  |  |
| Noggin Fw | GCCAGCACTATCTCCACATCCG |
| Noggin Rev | AGCAGCGTCTCGTTCAGATCCT |
|  |  |
| Musashi-1 Fw | GCTCAGCCAAAGGAGGTGATGT |
| Musashi-1 Rev | GCGTAGGTTGTGGCTTGGAAAC |
|  |  |
| Oct-3/4 Fw | CCTGAAGCAGAAGAGGATCACC |
| Oct-3/4 Rev | AAAGCGGCAGATGGTCGTTTGG |

Supplementary Table 3.

|  | **RC subunits** | **RC complexes** | **Mitochondrial respiration** | **ROS production** | **Affected protein network** | **MAM proteins** | **Mitochondrial calcium transients** |
| --- | --- | --- | --- | --- | --- | --- | --- |
|  |  |  |  |  |  |  |  |
| **Patient 1 Fibroblasts** | Normal | Normal | Normal | Normal | NT | Normal | **↓** |
| **Patient 2 Fibroblasts** | Normal | Normal | Normal | Normal | NT | Normal | Normal |
| **Carrier 1 Fibroblasts** | Normal | Normal | Normal | Normal | NT | Normal | NT |
| **Carrier 2 Fibroblasts** | Normal | Normal | Normal | Normal | NT | Normal | NT |
| **Patient 1 iNPCs** | MTCO1 **↓** | CI,CII,CIII,  CIV,CV **↓** | **↓** | **↓** | Mitochondrial metabolism  VAPB associated  Proteins | VAPB **↓**  PTPIP51 **↓** | **↓** |
| **Patient 2 iNPCs** | NDUFB8 **↓** | Normal | Normal | Normal | Mitochondrial metabolism  VAPB associated  Proteins  Fatty acid-beta oxidation | Normal | Normal |
| **Carrier 1 iNPCs** | Normal | Normal | Normal | Normal | Normal | Normal | NT |
| **Carrier 2 iNPCs** | NDUFB8 **↓** | Normal | Normal | Normal | Normal | Normal | NT |
| ***Gars^C201R/C201R^* Brain** | Normal | NT | NT | N/A | NT | Normal | N/A |
| ***Gars^C201R/C201R^* Liver** | Normal | NT | NT | N/A | NT | Normal | N/A |
| ***Gars^C201R/C201R^* Kidney** | Normal | NT | NT | N/A | NT | Normal | N/A |
| ***Gars^C201R/C201R^* Heart** | Normal | NT | NT | N/A | NT | Normal | N/A |
| ***Gars^C201R/C201R^* Muscle** | Normal | NT | NT | N/A | NT | Normal | N/A |
| ***Gars^C201R/C201R^* Sciatic nerve** | ATP5, UQCRC2,MTCO1,  SDHB,NDUFB8 **↓** | NT | NT | N/A | NT | VAPB **↓**  PTPIP51 **↓** | N/A |

**Legends for Supplementary Figures and Tables**

**Supplementary Figure 1.**

**Characterization of iNPCs by Quatitative RT-PCR and immunostaining.**

iNPCs and primary fibroblasts were collected and then subjected to total RNA extraction and

real-time PCR analysis. Quantitative Real-Time PCR in converted cells confirmed the efficacy of the transfection with oct3/4, sox2, kif4 and c-myc **(A-D**). Immunofluorescence staining of all iNPC cultures used in this study at day 20 reveals loss of Collagen I (fibroblast marker) and increased expression of iNPC marker Nestin and SB100in the iNPCs. DAPI was used to visualise nuclei. Scale bars, 20 μm **(E)**.

**Supplementary Figure 2. GO-term enrichment analysis of differentially expressed RNAs sequences in iNPCS cell lines.**

RNAseq data analysis compared RNA expression in patient versus control iNPC cells. GO-terms were significantly enriched for genes showing significant up (red bars) or down (blue bars) regulation of RNA expression. The most significant GO-terms, with the largest –Log10 Qvalues, differed between patient1 **(A)** and patient2 **(B)**.

**Supplementary Figure 3.**

**Mitochondrial DNA copy numbers and GARS protein levels in fibroblasts and iNPCs. (A)** Mitochondrial DNA copy numbers showed significant increase in iNPCs (n=3 experimental replicates), p values are from an unpaired t-test. Where indicated *p<0.05. Representative graphs display the expression level of GARS in fibroblasts and iNPCs **(B-E)**. Amount of GARS protein in the total cell lysate of fibroblasts **(B)**, mitochondrial fraction of fibroblasts **(C)**, total cell lysate of iNPCs **(D)** and mitochondrial fraction of iNPCs **(E).** One-way ANOVA followed by Bonferroni post hoc test (with 97% confidence intervals) was used for statistical analysis (*p<0.5 compared to control samples) (n=3 experimental replicates).

**Supplementary Figure 4.**

**BN-PAGE analysis of mitochondrial respiratory chain complexes in newborn (P0) *WT*, *Gars^C201R/wt^* and *Gars^C201R/C201R^* mouse brain, liver, kidney heart and muscle showed no significant abnormalities.**

**(A-E)** Mitochondrial complexes isolated from wt and mutant mice (n=5 in each group) were separated using blue native gel electrophoresis on gradient Bis-Tris acrylamide gels. To calculate the steady state levels of fully assembled complexes three independent blue native blots were quantified with band densitometry. Graphs represent the amount of respiratory chain complexes; the average numerical values were normalised to porin. Error bars represent standard deviation. **(F-I)** The activities of citrate synthase and complex II were measured by spectrophotometry. Complex IV activity was measured as reduction of cytochrome c at 550 nm. All measured parameters were expressed in specific activity per mg/protein in the sample, and related to the activity of citrate synthase. Total protein was quantified by Bradford assay.

**Supplementary Figure 5.**

**BN-PAGE analysis of mitochondrial respiratory chain complexes in 14 days old (P14) *WT, Gars^C201R/wt^ and Gars^C201R/C210R^* mouse brain, liver, kidney heart and muscle showed no significant abnormalities.**

**(A-E)** Mitochondrial complexes isolated from wild type and mutant mice (n=5 in each group) were separated using blue native gel electrophoresis on gradient Bis-Tris acrylamide gels. To calculate the steady state levels of fully assembled complexes three independent blue native blots were quantified with band densitometry. Graphs represent the level of complexes; the average numerical values were normalised to the porin levels. Error bars represent standard deviation. **(F-I).** The activities of citrate synthase and complex II were measured by spectrophotometry. Complex IV activity was measured as reduction of cytochrome c at 550 nm. All measured parameters were expressed in specific activity per mg/protein in the sample and were related to the activity of citrate synthase. Total protein was quantified by Bradford assay.

**Supplementary figure 6.**

**^35^S-methionine pulse labelling for mitochondrial translation in mouse fibroblasts.**

Mitochondrial translation products in isolated mouse fibroblasts from WT, heterozygous (*Gars^C201R/WT^* ) and homozygous (*Gars^C201RC210Rt^)* animals metabolically labelled with ^35^S for 15 minutes detected no defect of mitochondrial translation. Representative Coomassie brilliant blue- stained (CBS) gel was used as a loading control.

**Supplementary Table 1.**

**Differentially regulated proteins in patient iNPCs.**

Comparative proteomic analysis performed on 3 iNPCs (control, patient 1 and patient 2). Differentially expressed (>2 fold) proteins are highlighted with blue or red indicating up-, or downregulation respectively.

**Supplementary Table 2.**

**Primer sequences of genes confirmed with qRT-PCR (5` - 3`).**

**Supplementary Table 3. Summary of data collected from our study including cellular models (fibroblasts, iNPCs) and transgenic mouse model.** MTCO1=cytochrome C oxidase subunit 1; NDUFB8= NADH dehydrogenase [ubiquinone] 1 beta subcomplex subunit 8; ATP5= ATP synthase subunit 5; UQCRC2= Cytochrome b-c1 complex subunit 2; SDHB= Succinate dehydrogenase [ubiquinone] iron-sulfur subunit; CI= Complex I; CII= Complex II; CIII=Complex III; CIV=Complex IV; CV=Complex V; VAPB= Vesicle Associated membrane protein; PTPIP51= Regulator of microtubule dynamics protein 3; NT=Not tested; N/A=Not applicable;
